# Childhood, Adolescent and Young Adult Poor-Prognosis Rhabdomyosarcoma

**DOI:** 10.3390/cancers17193100

**Published:** 2025-09-23

**Authors:** Ajla T. Wasti, Gianni Bisogno, Raquel Hladun, Anne-Sophie Defachelles, Michela Casanova, Willemijn B. Breunis, Susanne A. Gatz, Reineke A. Schoot, Andrea Ferrari, Meriel Jenney, Rita Alaggio, Raquel Davila Fajardo, Sheila Terwisscha van Scheltinga, Janet Shipley, Michael Torsten Meister, Rick R. van Rijn, John Anderson, Monika Sparber-Sauer, Julia C. Chisholm, Johannes H. M. Merks

**Affiliations:** 1 Children and Young People’s Unit, The Royal Marsden NHS Foundation Trust, Sutton SM2 5PT, UK; ajla.wasti@nhs.net (A.T.W.); julia.chisholm@rmh.nhs.uk (J.C.C.); 2 Institute of Cancer Research, Sutton SM2 5NG, UK; 3 Children and Young People’s Cancer Services, University College London Hospitals NHS Foundation Trust, London NW1 2BU, UK; 4 Department of Women’s and Children’s Health, University of Padua, 35122 Padua, Italy; 5 Pediatric Hematology Oncology Division, University Hospital of Padua, 35128 Padua, Italy; 6 Pediatric Oncology and Hematology Department, Hospital Universitari Vall d’Hebron, Universitat Autònoma de Barcelona, 08035 Barcelona, Spain; 7 Pediatric and Adolescents/Young Adults Oncology Unit, Centre Oscar Lambret, 59020 Lille, France; 8 Pediatric Oncology Unit, Fondazione IRCCS Istituto Nazionale dei Tumori, 20133 Milan, Italy; 9 Department of Oncology and Children’s Research Center, University Children’s Hospital of Zürich, 8032 Zürich, Switzerland; 10 Department of Cancer and Genomic Sciences, College of Medicine and Health, University of Birmingham, Birmingham B15 2TT, UK; 11 Department of Paediatric Oncology, Birmingham Women’s and Children’s NHS Foundation Trust, Birmingham B4 6NH, UK; 12 Princess Máxima Center for Pediatric Oncology, 3584 CS Utrecht, The Netherlands; 13 Department of Oncology and Hemato-Oncology, University of Milan, 20122 Milan, Italy; 14 Department of Paediatric Oncology, Children’s Hospital for Wales, Heath Park, Cardiff CF14 4XW, UK; 15 Pathology Unit, Department of Laboratories, Bambino Gesu Children’s Hospital, IRCCS, 00165 Rome, Italy; 16 Department of Radiation Oncology, University Medical Center Utrecht, 3584 CX Utrecht, The Netherlands; 17 Division of Cancer Biology, The Institute of Cancer Research, Sutton SM2 5NG, UK; 18 Oncode Institute, 3521 AL Utrecht, The Netherlands; 19 Department of Radiology and Nuclear Medicine, Amsterdam UMC, University of Amsterdam, 1105 AZ Amsterdam, The Netherlands; 20 Developmental Biology and Cancer Programme, University College London Great Ormond Street Institute of Child Health, London WC1N 1EH, UK; 21 Klinikum der Landeshauptstadt Stuttgart gKAöR, Olgahospital, Stuttgart Cancer Center, Zentrum für Kinder-, Jugend- und Frauenmedizin, Pädiatrie 5 (Pädiatrische Onkologie, Hämatologie, Immunologie), 70174 Stuttgart, Germany; 22 Department of Pediatric Hematology and Oncology, University Children’s Hospital Tuebingen, 72076 Tuebingen, Germany; 23 Division of Imaging and Oncology, University Medical Center Utrecht, Utrecht University, 3584 CX Utrecht, The Netherlands

**Keywords:** rhabdomyosarcoma, metastatic, relapse, child, adolescent, young adult, chemotherapy, radiotherapy, surgery, novel approaches

## Abstract

Rhabdomyosarcoma (RMS) is the most common soft tissue sarcoma in children and young people. Despite the advances in multimodality treatment over recent decades through successive prospective clinical trials, improved rates of survival for patients are mainly limited to those with localised RMS without adverse biologic features. Current clinicopathologic prognostic factors include *PAX3(7)::FOXO1* fusion status, the site of primary disease, the pre-chemotherapy extent of disease (including microscopic vs. macroscopic residual disease, locoregional nodal involvement and metastatic status), tumour size and patient age. These factors are used to stratify patients into prognostic risk groups that guide treatment intensity and duration. Risk stratification algorithms are evolving, supported by advances in molecular biology and cancer genomics. In this review we focus on the poorest prognostic groups of paediatric-type RMS (i.e., Very High Risk or relapsed/progressive disease). These include patients whose tumours harbour poor biological characteristics such as *PAX3(7)::FOXO1* fusion-positive tumours with locoregional nodal involvement and tumours harbouring other poor-risk genetic variants (particularly *MYOD1* and *TP53* variants); adolescent and young adult patients; newly diagnosed patients with metastatic RMS; and patients with relapsed and refractory disease. Here we aim to describe the clinical characteristics of these patients, outline current standard multimodality treatments in the context of sequential international clinical trials across the major cooperative groups and summarise emerging novel diagnostic and therapeutic approaches.

## Simple Summary

Rhabdomyosarcoma (RMS) is an aggressive soft tissue sarcoma which primarily affects children, adolescents and young adults. Multimodality approaches to treatment include chemotherapy, surgery and radiotherapy. Despite attempts to improve outcomes over recent decades, survival rates remain poor for patients with advanced and relapsed/progressive disease and those who have tumours with aggressive biologic features. This review article focuses on patient groups with the poorest outcomes which include those with (1) *PAX3(7)::FOXO1* gene fusions and other adverse biological features; (2) RMS in adolescents and young adults; (3) RMS that has already spread to distant organs at the time of first presentation (metastatic disease); (4) RMS that progresses or recurs following first-line treatment. This review highlights the ongoing urgent unmet need for improved treatments in these patients and discusses novel therapeutic approaches currently being investigated.

## 1. Introduction

Rhabdomyosarcoma (RMS) is a mesenchymal neoplasm with morphologic and immunophenotypic features of skeletal muscle differentiation [1]. It is the most common soft tissue sarcoma (STS) in children and young people (CYP) accounting for about 3% of all childhood cancers and remains a major contributor to cancer-related deaths [2,3]. Despite the advances in multimodality treatment over recent decades through successive prospective clinical trials executed by international paediatric oncology consortia, improved rates of survival for patients are mainly limited to those with localised RMS without adverse biologic features. Current clinicopathologic prognostic factors, which include *PAX3(7)::FOXO1* fusion status, the site of primary disease, tumour size, patient age, the extent of pre-chemotherapy disease and locoregional nodal involvement, are well recognised in localised RMS and have been used in clinical trials to stratify patients into prognostic groups that guide treatment intensity and duration. Although these risk factors are similar amongst collaborative groups, they are used slightly differently by different consortia resulting in non-uniform risk stratification algorithms. Risk stratification evolves with an improved understanding of how clinical and biologic features affect disease course; advances in molecular biology and cancer genomics are enabling more sophisticated biologically based risk stratification systems. Table 1 shows the current European *paediatric* Soft Tissue Sarcoma Group (E*p*SSG) risk stratification for patients with rhabdomyosarcoma and reports EFS and OS from RMS 2005 and MTS 2008 [4,5,6].

In this review we will primarily focus on the poorest prognostic groups of paediatric-type RMS (subsequently termed poor-risk disease) which include (i) newly diagnosed patients who have biologically poor-risk non-metastatic disease, particularly *PAX3(7)::FOXO1* fusion positive (FP) with concomitant lymph node positivity (N1) (5-year overall survival (OS): 45.5%; 5-year event-free survival (EFS): 43%) [7]; newly diagnosed patients with tumours harbouring poor-risk genetic variants (*MYOD1* or *TP53*) or more recently identified fusions (*FUS/EWSR1::TFCP2*, *MEIS1::NCOA2* or *ZFP64::NCOA3*) [8,9,10]; (ii) adolescent and young adult (AYA) patients; (iii) newly diagnosed patients with metastatic disease (3-year OS: 49.3%; EFS: 35.5%) [11,12]; (iv) patients with relapsed and/or refractory disease (3-year OS: 17%) [12]. Here we aim to describe their clinical characteristics, outline different aspects of current standard multimodality treatments and their historic implementation across cooperative groups and hypothesise how this may evolve to improve outcomes in the future.

## 2. Biologically Poor-Risk Disease

Histology as a surrogate for adverse biology has long been included in RMS risk stratification systems. Advances in our understanding of tumour biology and the availability of molecular pathology testing techniques and data have enabled us to further recognise underlying molecular risk factors of disease. The current fifth World Health Organization (WHO) histological classification of RMS includes embryonal rhabdomyosarcoma (ERMS), alveolar rhabdomyosarcoma (ARMS), spindle cell/sclerosing rhabdomyosarcoma (ssRMS) and pleomorphic rhabdomyosarcoma (almost exclusively seen in adults and not considered in this review) [13,14]. However, it is now well recognised that certain molecular characteristics are better prognostic biomarkers than histology. Two dominant molecular subtypes exist in RMS: *PAX3(7)::FOXO1* FP and *PAX3(7)::FOXO1* fusion-negative (FN) RMS, most usually ARMS and ERMS, respectively. Among patients with ARMS, approximately 80% are characterised by the *PAX3* or *PAX7::FOXO1* fusion [14]. Apart from the characteristic translocations giving rise to these fusions, the burden of somatic aberrations in FP RMS is relatively low [15]. In contrast, FN RMS often harbours aberrancies in key signalling pathways and/or other rarer mutations and gene fusions [14,15,16,17]. An improved understanding of specific molecular landscapes can help inform adaptations to current risk stratification systems and also potentially indicate biologically based novel therapeutic strategies. In addition to *PAX3(7)::FOXO1* fusion status, we will briefly discuss other poor prognosis molecular subgroups.

### 2.1. PAX3(7)::FOXO1 Fusion-Positive RMS

FP RMS harbours either a characteristic t(2;13)(q35;q14) translocation giving rise to the fusion of *PAX3* with *FOXO1* (60% of cases) or a t(1;13)(p36q14) translocation resulting in the fusion of *PAX7* and *FOXO1* (20% of cases) [1]. *PAX3* and P*AX7* code for a family of transcription factors expressed in skeletal muscle progenitor cells and *FOXO1* codes for a forkhead transcription factor. The gene fusions encode a novel aberrant transcription factor with a DNA-binding domain at the amino terminus of the PAX3 or PAX7 protein and a transcriptional activation domain at the carboxyl terminus on FOXO1 [1]. Enhanced transcriptional activation is thought to be a functional result of the decreased sensitivity of *FOXO1* to inhibitory effects of *PAX3* or *PAX7* and also a result of higher levels of mRNA and protein expression of *PAX3(7)::FOXO1* [1]. *PAX3::FOXO1* has been more extensively studied and as a transcription factor increases the expression of downstream target genes by binding to *PAX3*-binding sites in close proximity to these genes. It also reprogrammes the chromatin landscape, enabling target gene promotor initiation [1]. *PAX3::FOXO1* and *PAX7::FOXO1* therefore functionally act as oncoproteins through their dysregulation of multiple downstream cellular networks that promote cellular phenotypes such as increased growth, survival and differentiation [1]. Alternate rare fusions with *PAX3* have been reported, but there are limited outcome data on patients with these molecular subtypes and, to date, no definitive clinical relevance can be ascertained.

*PAX3(7)::FOXO1* fusion positivity is recognised as one of the strongest negative prognostic factors in RMS. Evidence to support the use of fusion status instead of morphology in risk stratification comes from the finding that 20% of ARMS cases that are FN have gene expression profiling, disease course and outcomes more in keeping with ERMS [17,18,19,20,21]. *FOXO1* fusion status has replaced favourable or unfavourable histology in current RMS risk stratification systems in the Children’s Oncology Group (COG) and E*p*SSG [4,22]. This is an important example of the use of molecular diagnostic tools to guide risk group allocation in this new era of molecular disease characterisation in paediatric oncology [23]. The recently completed COG trial ARST1431 and currently ongoing E*p*SSG FaR-RMS (Frontline and Relapsed RMS) trial prospectively evaluate this modified risk stratification which de-escalates therapy for alveolar, FN patients with localised disease [4,24].

Evidence for whether the *FOXO1* fusion partner *PAX3* or *PAX7* differentially impacts prognosis has thus far been equivocal [25]. Publications from the COG have shown that in metastatic patients, those with *PAX3::FOXO1* gene fusions had poorer outcomes and a higher correlation with bone marrow involvement [26]. In a univariate analysis of patients with localised disease, those with *PAX7* had an improved prognosis [27]. Currently both translocations are considered unfavourable in clinical risk stratification systems.

Accompanying genetic aberrations in FP tumours most commonly include focal *CDK4* (13%) and *MYCN* (10%) amplifications while variants in *BCOR*, *NF1*, *TP53* and *PIK3CA* are less frequent [15]. The additive impact of these secondary aberrations on clinical course remains to be elucidated. The amplification of 1p36 and 13q13, which contain *PAX7* and *FOXO1* genes, respectively, are seen in 90% of ARMS with a *PAX7::FOXO1* fusion [28]. *PAX7* amplification in *PAX7::FOXO1* ARMS has been associated with an improved prognosis, which has not been confirmed by multivariate analysis [28]. The amplifications of 2p24 (containing *MYCN*) in approximately 10–15% of ARMS and 12q13-q14 (containing *CDK4*), most commonly seen in *PAX3::FOXO1* fusions, may be associated with a worse prognosis, but this needs to be confirmed in larger studies [29]. Somatic *TP53* mutations are found in <4% of ARMS and are associated with a poor prognosis [10].

Non-metastatic ARMS with regional nodal involvement accounts for about 10% of all newly diagnosed patients with RMS [7,30]. In the E*p*SSG RMS 2005 trial, the 5-year EFS of patients with FP ARMS N1 tumours was significantly inferior to that of patients with FN ARMS N1 tumours (43% vs. 74%, *p* = 0.01) underscoring the prognostic superiority of molecular subtype over histologic subtype [7]. Irrespective of the primary site or the chemotherapy regimen used to treat FP N1 RMS, patient outcomes are similar [31]. The poor EFS and OS, which are only marginally better than those for metastatic RMS, have influenced the current E*p*SSG approach to stratify N1 FP RMS into the Very High-Risk (VHR) group which also includes patients with metastatic disease (M1), resulting in more intensive treatment [4]. The ability to discern between *PAX3(7)::FOXO1* FP and FN disease has enabled a firm embedding of this molecular variable into current risk stratification systems both in the E*p*SSG as well as COG and allows for improved accuracy in identifying RMS subtypes of clinical significance. Fusion status however is only the first of several molecular biomarkers in RMS that are, or in the near future will be, incorporated into risk stratification systems and exemplifies the dynamic process by which prognostic factors are considered.

### 2.2. PAX3(7)::FOXO1 Fusion-Negative Poor-Risk RMS Subtypes

Fusion-negative RMS tumours most often have an embryonal histology, are aneuploid and can harbour molecular aberrations such as a loss of heterozygosity (LoH) at 11p15, with chromosomal gains (2, 8, 7q, 11q and 13), losses (10 and 15) and mutations of *TP53*, *PIK3CA*, *FGFR4*, *FBXW7*, *ALK*, *BCOR*, *NF1*, *CTNNB1*, *ARID1A*, *MYOD1*, *CDKN2A* and *CDKN2B* [10,23,32,33,34]. Mutations in the RAS pathway are the commonest aberration in FN RMS, present in >50% of cases, with an even higher predominance in infants [10,23]. RAS mutations currently are not considered on their own to convey poorer outcomes [10,23] although previous smaller retrospective molecular studies had suggested this [35,36]. The clinical and prognostic significance of several candidate mutations is currently under investigation. How RMS patients with specific molecular features should be treated remains under debate, with some proposing the “upstaging” of certain biological subgroups to justify more aggressive treatment [4,22,23].

#### 2.2.1. *TP53* Variants

*TP53* induces the transcription of genes associated with various cellular activities including cell cycle arrest, apoptosis and cellular metabolism [37]. *TP53* also has transcription-independent properties such as a role in DNA repair [38]. Molecular variants that are functionally tumour suppressors, loss-of-function mutations, deletions or gain-of-function mutations in *TP53* have been found in several cancers, including RMS, often resulting in the loss of tumour suppressor function and/or gain of oncogenic properties [39,40,41,42]. *TP53* is the key germline cancer predisposition gene implicated in Li–Fraumeni Syndrome (LFS). Broadly there exist two types of *TP53* mutations: the first in which mutations occur at amino acids directly interacting with DNA, and the second in which mutations change the conformational structure of *TP53*, resulting in a loss of DNA binding activity [40,41,43,44,45]. In a recent analysis of various solid tumours (including RMS) from patients with LFS, somatic mutations implicating Wnt and PI3K/AKT pathways, epigenetic modifications, as well as homologous recombination and “prior chemotherapy” specific mutational signatures were observed [46]. Furthermore, an earlier LoH of *TP53* and gain of the mutant allele may distinguish germline from somatic *TP53* mutations [46].

Clinically, patients with RMS harbouring *TP53* mutations comprise a heterogenous group. Mutations may be germline or somatic and occur in FN and FP RMS. In all cases, the aggressive clinical course of patients with *TP53* mutations has been established. Shern, Selfe and colleagues performed an international consortium study designed to determine the incidence of driver mutations and their association with the outcome in RMS. Tumour sample DNA was collected from 631 patients enrolled on COG, International Society of Pediatric Oncology (SIOP) Malignant Mesenchymal Tumour (MMT) and E*p*SSG (RMS2005) studies between 1995 and 2017. Through custom capture sequencing, deep deletions, truncating mutations and point mutations were found throughout the mutant *TP53* genes with some enrichment seen within the DNA-binding domain. Although we do not know yet whether specific mutations in the *TP53* gene confer different prognoses, this analysis revealed the incidence of *TP53* mutations to be higher than previously reported at 13% (69/515) in FN RMS and 4% (5/126) in FP RMS [10]. Due to a lack of matched germline samples, the authors were unable to ascertain whether the lesions represented somatic or germline events. *TP53* mutations were associated with an inferior OS and EFS in both COG and SIOP-MMT/E*p*SSG cohorts (in both univariate and risk-stratified analyses) with authors nominating alterations in *TP53* as a poor prognostic biomarker in both FN and FP RMS [10]. Although there is accumulating evidence that *TP53* variants may be an independent adverse prognostic factor, *TP53* status has not yet been uniformly adopted into risk stratification schema across collaborative groups. Whereas the E*p*SSG has not implemented change based on *TP53* status, the COG has proposed that patients with very low-risk (VLR) or low-risk (LR) disease must have wild-type *TP53* (and *MYOD1*) [47].

In a study by Shenoy et al., tumour mutational data (based on targeted panel sequencing) and central pathology review was ascertained for 146 patients (in whom *TP53* status was available) enrolled on a series of COG studies between 1997 and 2013 [48]. *TP53* mutations were found to be present in 9% of tumours (13/146). Among the 38 cases of anaplastic RMS in this patient cohort, 24% (9/38) had *TP53* mutations. The majority of *TP53* mutant tumours were noted to have an anaplastic histology (9/13; 69%) [48]. The study refuted previous reports that anaplasia was an independent poor prognostic factor but the authors concluded that in future, the presence of anaplasia could be used to identify tumours with the highest likelihood of harbouring a *TP53* mutation, highlighting again that molecular subgrouping outperforms histologic subtype as a prognostic biomarker in RMS [48].

Hettmer et al. looked at RMS tissue from a small number of patients with germline *TP53* mutations to determine whether this predisposed patients to develop RMS with anaplastic histology [49]. Eight cases with known *TP53* germline mutations and RMS had their tumour histology retrospectively reviewed and seven cases of anaplastic RMS with unknown germline *TP53* status underwent germline testing. Eleven *TP53* germline mutation carriers with a median age of 40 months at diagnosis all exhibited an non-alveolar anaplastic histology. Among patients with LFS, anaplastic RMS may soon be recognised as one of the sentinel cancers indicating the need for germline *TP53* mutation screening irrespective of age or family history [49]. Referral to genetics for germline mutation testing of several potential cancer predisposition genes is recommended for children with RMS who are younger than 5 years of age at the time of initial diagnosis, so that early identification can result in surveillance screening and the earlier detection of second malignancies for at-risk populations [50,51].

#### 2.2.2. MYOD1 Variants

RMS harbouring a *MYOD1*^L122R^ variant can be found in patients of all ages although it is slightly more common in adults and older children [52]. *MYOD1*-mutant tumours frequently involve the head and neck extremities and trunk and have a slight female preponderance [52]. A retrospective analysis of pooled paediatric RMS cases from the US and UK showed a frequency of 3% among *PAX3(7)::FOXO1* FN tumours (17/515) and these patients had universally poor outcomes, independent of clinical risk stratification [10]. The subtype of ssRMS in the current WHO classification includes RMS with *MYOD1* mutations, although the mutation has been identified in ERMS also (less frequently). Since the initial recognition of ssRMS as an entity in the 2013 WHO classification, tumours harbouring *MYOD1* mutations have been identified as a distinct poor prognostic subset [53]. Studies interrogating both spindle cell and sclerosing RMS in children and adults have found homozygous or heterozygous mutations in exon 1 of *MYOD1*, almost exclusively p.L122R, further justifying their shared grouping in the WHO classification as a single entity [54]. Tumours harbouring *MYOD1* mutations have a more aggressive disease course and a less favourable prognosis, frequently progressing or recurring (often with metastases) despite the use of intense multimodal therapies [33,55]. In a recent review of 72 published cases of *MYOD1*-mutant RMS, only 12 of 37 patients (33%) with at least one year of follow-up were alive without disease and 43% had died [8]. A detailed analysis of 30 cases of *MYOD1*^L122R^-variant RMS showed that the median time to event in 25 patients with adequate follow-up data was 9 months and the majority (80%) of events included the failure of local control [52].

*MYOD1* is necessary for myogenic differentiation via the control of the MAPK and PI3K-AKT pathways. It encodes a nuclear protein that transcriptionally regulates the differentiation of muscle cells by inducing cell cycle arrest, which is required for myogenic initiation. Mutant *MYOD1* competes with wild-type *MYOD1* for binding sites conferring decreased transcriptional activation and cellular differentiation and also binds to sites regulated by *MYC* [56]. For a detailed discussion on the mechanism of mutant MYOD1, please see Di Carlo et al. 2023 [8].

Tumours with *MYOD1* mutations may also harbour additional co-mutations (*HRAS*, *NRAS*, *FGFR4*, *PIK3CA*), amplifications (*MYOD1*, *FGFR4*, *IGF2*, *MDM2*) or deep deletions (*PTEN*, *GATA3*, *CDKN2A/B*) and these too may impact prognosis although larger scale prospective studies are needed to explore this further [55,56,57].

In current clinical practice, the detection of somatic *MYOD1* mutation now leads to “upstaging” within the risk stratification schema both in the COG and E*p*SSG [8,47].

### 2.3. PAX3(7)::FOXO1 Fusion-Negative Newly Emerging RMS Subtypes of Uncertain Prognostic Significance

#### 2.3.1. TFCP2- and MEIS1-Associated Gene Fusions

RMS with *TFCP2*-associated gene fusions and less commonly *MEIS1::NCOA2* fusions are an aggressive, extremely rare and newly discovered entity of primary intraosseous RMS [58,59,60]. These tumours mostly affect young adult patients (median age at diagnosis of 31 years), have a slight female preponderance and are occasionally found in the soft tissue but are most often intraosseous and have a strong predilection for the craniofacial bones where they can often destroy the cortical bone and invade surrounding soft tissue. The median survival of patients with *TFCP2* fusion-positive RMS is poor (8 months in the largest published series) [9]. These tumours have a hybrid spindle cell and epithelioid histology and display nuclear pleomorphism and frequent mitoses. Underpinning this subtype is the *TFCP2* (12q13.12) gene rearrangement wherein the 5’ portion of the gene is fused to an *FET* partner (*EWSR1* or *FUS*) resulting in oncogenesis via transcriptional dysregulation through novel fusion proteins, the disruption of normal cellular functions and genomic instability [9,61,62,63,64,65].

*TFCP2* codes for an evolutionarily conserved transcription factor targeting thymidylate synthase involved in the enzymatic regulation of DNA synthesis and contains an N-terminus DNA-binding domain and a C-terminus sterile alpha motif domain that plays a role in protein dimerization [9]. Furthermore, these tumours have complex genetic profiles but have homogenous transcriptomes. In the largest published series of 14 cases of *FET::TFCP2*-positive tumours, all cases that underwent genomic profiling showed homozygous deletions of the *CDKN2A* tumour suppressor gene, while other homozygous deletions were also displayed in a small number of cases [9]. Alterations of *ALK* (without underlying *ALK* fusions) resulting in *ALK* overexpression at both the transcriptional and protein levels have also been noted in a number of cases [9,64]. The upregulation of *ALK* does not result from translocation or amplification but rather correlates with *ALK* genomic deletion and therefore may involve alternative transcription initiation [9]. *ALK* expression related to intragenic deletions and aberrant splicing results in oncogenic ALK variants [64]. Functional studies show that *FUS::TFCP2* blocks myogenic differentiation, induces the transcription of *ALK* and truncated *TERT*, and inhibits DNA repair [58]. *TFCP2*-rearranged tumours exhibit genomic instability and DNA methylation profiling demonstrates a close relationship with undifferentiated sarcomas [65]. Reports of sarcomas preceded by benign lesions carrying *FUS::TFCP2* indicate stepwise sarcomagenesis [59]. Le Loarer and colleagues suggest that *ALK* upregulation in their cohort was as high or higher than that found in *ALK*-rearranged tumours such as *ALK* fusion-positive inflammatory myofibroblastic tumours. Whether there is a subset of these patients in whom *ALK* inhibition could potentially be utilised as a component of therapy remains to be seen and further information is needed to understand how to better manage these patients [9].

#### 2.3.2. ZFP64::NCOA3 Gene Fusions

Han and colleagues have recently proposed a novel rare and aggressive molecular entity among spindle cell RMS which harbours a *ZFP64::NCOA3* gene fusion [66]. The group describes a retrospective archival analysis of tissue, yielding five cases all occurring in adult males (aged 28–71 yrs). In the reported cases, all tumours arose from deep soft tissues and three out of five developed distant metastases, two of whom died of disease within 24 months from initial diagnosis. Only one of the five patients received systemic chemotherapy with curative intent in the frontline setting. Histologically, these tumours lack rhabdomyoblasts and are made of spindle cells arranged in a herringbone pattern amidst collagenous to myxoid stroma with monomorphic nuclei and variable mitotic activity [66]. In two of the five cases identified, the foci of metaplastic bone or dystrophic calcification were present. Most tumours retained immunohistochemistry in keeping with rhabdomyoblastic differentiation (patchy staining for desmin and focal staining for MyoD1 but negative for myogenin) [66]. The authors propose this as a new entity among spindle cell RMS but admit that in the absence of prototypic rhabdomyoblasts, this molecular subgroup could also represent an aggressive myofibroblastic derivative and additional studies are needed to further characterise this subgroup of tumours. *Nuclear receptor coactivator 3* (*NCOA3*) is a member of the steroid receptor coactivator p160/SRC family and contributes to the pathophysiology of several cancers through gene expression modulation [67,68,69]. Zinc finger (ZF) proteins as a group are implicated in cell proliferation, apoptosis, immune function and tumorigenesis. *ZFP64* is a coactivator of Notch1 signalling and may mediate differentiation in mesenchymal cells [70].

With respect to novel fusions such as *PAX3::NCOA2* which have recently identified, it is not yet clear what the prognostic significance of these entities are. A recent publication suggests that this entity clusters with ARMS and *PAX3::MAML* on methylation profiling and therefore may be regarded like ARMS [71].

As we continue to identify poor-risk biologic candidates, the further refinement of E*p*SSG risk stratification is highly likely and follows a similar refinement within the COG [47].

## 3. Clinically Poor-Risk Disease

### 3.1. Rhabdomyosarcoma in Adolescents and Young Adults

RMS is typically a tumour of childhood but can occur at any age. Among the different prognostic factors, age in itself has been recognised as independently influencing survival, and as a consequence paediatric protocols include age as one of the variables used for risk stratification [72].

The survival rates of adolescent patients are inferior to those of children, but the outcome is even worse in adults, with historical published series reporting 5-year OS rates in the 20–40% range [73,74,75]. The reasons for this survival gap are likely multifactorial [75]. However, it has been suggested that the adverse outcome of AYA with RMS may be correlated, at least in part, to differences in clinical management (that could include patient referral patterns, time to diagnosis, enrolment into clinical trials and the adequacy and intensity of treatment). Various studies have suggested that AYA patients with RMS may have better outcomes when treated with multidisciplinary treatment in line with the paediatric approach, in particular with regard to the use of intensive multidrug chemotherapy [76,77,78,79,80,81]. However, outcomes seem to be inferior in AYA patients when compared with children, even when they were treated in the same way. A recent analysis from the E*p*SSG (on 1977 patients registered on two concurrent clinical protocols, the E*p*SSG RMS 2005 for localised RMS patients and the E*p*SSG MTS 2008 protocol for metastatic patients) reported a 5-year OS of 57.1% for patients aged 15–21 years and 77.9% for those aged 0–14 years old enrolled in the same trials (*p*-value < 0.0001) [80]. These data suggest that the more recent outcomes for AYA patients with RMS are better than those reported in epidemiological studies (for example, the EUROCARE-5 study which covered 2000–2007 showed a 39.6% 5-year OS in 15–19 year old patients), supporting the idea that these patients should be included in paediatric RMS trials to offer them the best chances of a cure. Poor AYA outcomes may suggest that part of the prognostic gap between children and AYA patients with RMS could be related to differences in tumour biology and the intrinsic aggressiveness of the disease. We discussed earlier in this manuscript the prognostic impact of RMS with *MYOD1* mutation and *TFPC2* gene fusions, both of which may have a higher incidence in the AYA age group when compared with younger age groups. In addition, an alveolar histology (as a surrogate for *PAX3(7)::FOXO 1* fusion status) forms a higher proportion of RMS cases in adolescent patients aged 15–21 years than in patients < 15 years (46% vs. 26%) [78]. Age-independent scientific collaboration and the development of more integrated genomic approaches focused on AYA patients are needed. While these are awaited, we estimate that RMS in AYA patients should be considered “poor-risk” in itself.

### 3.2. Patients with Metastatic (M1) Disease at Presentation

Approximately 15–20% of paediatric patients have at least one site of distant metastatic disease detected radiographically or clinically at the time of initial presentation. Table 2 summarises potential sites of metastatic disease and modalities which can be used in detection as part of staging. Metastatic spread occurs via both the haematogenous and lymphatic routes. The most common metastatic site is the lung (involved approximately 50% of the time) [11,81,82]. Bone marrow, bone (each involved around 30% of the time) and distant nodes are also common sites of metastatic involvement with rarer sites including pleura, peritoneum and cerebrospinal fluid (CSF) [82]. Outcomes for patients with metastatic disease remain dismal and although most patients achieve an objective response with systemic induction chemotherapy (92% of patients achieved ≥33% volume reduction in the primary tumour) and local control measures, the majority of patients relapse, resulting in a historic 3-year EFS and OS of only 27% and 34%, respectively [6,82]. As for adolescents and adults there is an overlap between clinical and biological risk factors: in recent European studies 57–60% of patients with metastatic RMS have an alveolar histology compared with 24% of patients with localised RMS [5,80,83]. For this group of patients, frontline treatment has not advanced significantly over the last several decades in spite of attempts at therapy intensification; new approaches are desperately needed.

#### 3.2.1. Risk Stratification in Metastatic Disease

The presence of distant metastases at diagnosis is the single most powerful predictor of survival in patients with RMS. An analysis of 788 patients enrolled on European and COG studies between 1984 and 2000 aimed to define pretreatment clinical characteristics that could stratify patients into risk groups to guide treatment decisions, such that patients with the highest risk of relapse could be identified for frontline innovative therapies [82]. Four independent adverse risk factors (Oberlin factors) were identified: age < 1 year or ≥10 yrs, site (extremity, other or unknown primary), bone or bone marrow involvement and ≥3 metastatic organ sites. The 3-year EFS was 50% for patients without any risk factors and 42%, 18%, 12% and 5%, respectively, in patients with one, two, three or four risk factors [82]. Two distinctly different prognostic subgroups were identified through this analysis: patients with 0–1 Oberlin risk factors (3-year EFS of 44%) and those with 2–4 Oberlin risk factors (3-year EFS of 14%) delineating favourable and unfavourable sub-strata within metastatic RMS [82]. However, outside the trial setting, no cooperative group is currently uniformly using this approach to risk stratify patients or inform treatment decisions in patients with metastatic RMS. Within the current E*p*SSG FaR-RMS study, Oberlin risk factors are taken into account to establish eligibility for radiotherapy (RT) randomisations [4]. Notably, histology was not found to be a significant prognostic factor in the Oberlin analysis and *PAX3(7)::FOXO1* fusion status was not available for the patients included in the analysis. In the future, a similar analysis, taking into account adverse biologic features such as fusion status may provide novel insights.

The recently published E*p*SSG MTS 2008 study in metastatic RMS analysed outcomes by Oberlin risk factors and showed that patients with 0–2 Oberlin risk factors have an improved 3-year OS (60%) compared with the historical Oberlin cohort [6]. This may be the result of the gradual introduction of more sensitive staging diagnostics, potentially adding, for instance, a site to the cumulative Oberlin risk factors and thereby allowing for the more accurate calculation of risk for some patients [6].

In a recently published COG paper, pooled data from six COG trials (three of which included metastatic patients; *n* = 187) was used for survival tree regression for EFS to recursively select prognostic factors that would result in the branching and split of the data [84]. In this analysis, the clinical factors included were age, *FOXO1* status, clinical group, histology, nodal status, the number of metastatic organ sites, primary site, sex, tumour size and the presence of metastases in specific sites. In patients with metastatic RMS, *FOXO1* FP was the strongest negative prognostic factor [84]. Five-year EFS and OS were 46% and 58%, respectively, for FN patients and only 6% and 19%, respectively, for FP patients [84]. Their model identified metastatic FN patients with a single site of metastatic disease as a better prognostic group with improved 5-year EFS (54%) and OS (70%) compared with FN patients with >one site of metastatic disease (5-year EFS of 34% and OS of 40%). The authors proposed a high-risk group which would include all metastatic patients with FP RMS and metastatic patients with FN RMS who had more than one metastatic site [84]. The latter group, i.e., single-organ site metastatic disease has been reported in multiple studies to have higher rates of survival [82,84,85,86,87].

The COG study ARST0431 showed that among metastatic patients, the group (*n* = 20) that appeared to have an improved OS were patients with embryonal RMS who were younger than 10 years old at the time of initial diagnosis, carrying a 3-year EFS and OS of 60% and 79%, respectively [88]. It is on this basis that within the COG, metastatic patients younger than 10 years with FN RMS are considered a more favourable prognostic subgroup and are treated as Intermediate Risk (IR). By contrast, within the E*p*SSG and Cooperative Weichteilsarkom Studiengruppe (CWS), fusion status or age does not currently impact how metastatic patients are risk stratified or treated, as all metastatic patients are stratified as VHR within the current FaR-RMS study.

Patients with distal extremity RMS who present with proximal nodal involvement (without distant metastatic disease) have a prognosis comparable to M1 patients [89,90,91]. In contrast, patients with distal extremity primaries with only popliteal or epitrochlear nodal involvement have comparatively better outcomes [91]. An interesting point of discussion was broached by Terwisscha et al. exploring the idea of considering patients with distal extremity RMS and proximal nodal involvement as having the equivalent of metastatic disease given their poor prognosis [91]. Although this specific approach has not yet been adopted, it is important to note that the majority of patients with distal extremity RMS were found to have an alveolar histology (and when tumours were tested, were FP) and this subgroup (FP N1) is known for poor outcomes and has been grouped together with metastatic disease in E*p*SSG’s current VHR group in the FaR-RMS trial. Perhaps the small subgroup of FN cases in this cohort requires prospective validation to understand this further.

#### 3.2.2. Single-Organ Metastatic Involvement

The commonest single metastatic site in RMS is the lung followed by the lymph nodes [82,86]. A number of studies suggest improved outcomes in lung-only metastatic RMS [86,87]. By definition, single-organ metastatic disease involving a site other than bone or bone marrow can at most have two Oberlin risk factors which may contribute to improved subgroup outcomes.

A COG study recently analysed 428 patients with metastatic ERMS and ARMS. The 55 patients with lung-only metastases were more likely to be <10 years of age or have ERMS and were less likely to have locoregional nodal disease or primary extremity tumours [86]. The lung-only cohort had significantly better survival outcomes than patients with all other sites of metastases with a 5-year EFS of 48.1 versus 18.8% and a 5-year OS of 64.1 versus 26.9% [86]. In patients with ERMS and lung-only metastases, there was no significant difference in survival between patients ≥ 10 years and 1–9 years, raising the question of whether all patients irrespective of age with FN RMS and lung-only metastases will be stratified differently in future COG studies [86].

An E*p*SSG analysis of 270 M1 patients registered on the MTS 2008 study reported that among 59 patients with lung-only metastases, the 3-year EFS and OS were 40% and 60%, respectively, compared with 28% and 35% in the lung and other sites and 36% and 49%, respectively, in other single organ sites [87]. The improved outcomes in lung-only metastatic RMS could be accounted for by these patients having fewer Oberlin risk factors than other metastatic patients [87].

The CWS performed a similar analysis, although their patient cohort was restricted to 53 patients with ERMS and lung-only metastases and the authors found that the 5-year EFS and OS were 41% and 52%, respectively. This improved outcome is expected given that the analysis was restricted to favourable histology/fusion-negative patients with single-organ metastases [92].

Mercolini et al. investigated the clinical characteristics and outcomes of M1 patients on E*p*SSG protocols in whom distant lymph nodes were the only site of metastatic involvement, accounting for about 7% of all metastatic patients. In 22 eligible patients the primary tumour was often ARMS (*n* = 15) and/or >5 cm in size and/or was located in the distal extremity. Patients with metastatic nodal involvement almost always had locoregional nodal involvement (21/22) [85]. Of the 18 tumours for which the fusion status was available, 12 were FP. Following the completion of induction chemotherapy and local control (surgery in 50% and RT in 91%) most patients went on to receive maintenance chemotherapy. The median follow-up was 54 months and the 3-year EFS and OS were 67.1% and 71.9%, respectively [85]. In the cohort of patients for whom fusion status was known, no events occurred in the six patients with FN RMS and the 3-year EFS and OS for FN vs. FP patients were 100% and 100% vs. 46.6% and 57.1%, respectively [85]. The results of this study suggest that this patient subgroup, and especially the FN patients, may represent a better prognostic subgroup among metastatic patients. The 3-year EFS and OS were 72% and 79.4%, respectively, for patients with zero or one Oberlin risk factors and 57.1% and 57.1% for those with two factors, which are markedly improved according to what would have been predicted by Oberlin et al. from the 2008 retrospective analysis [82]. The authors hypothesise that lymphatic dissemination is likely to be biologically distinct from haematogenous spread and this may impact improved survival in this patient subgroup. They went on to further postulate that the comparative ease with which local control can be delivered (in particular with the use of RT) to lymph nodes may also positively impact survival rates [85].

The result of this study raises the question of whether RMS patients with distant node-only metastatic disease should continue to be treated in the same way as other metastatic patients. Given the limited sample size, future initiatives aimed at addressing this question through the analysis of pooled data in the International Soft Tissue Sarcoma Consortium (INSTRuCT) database are currently ongoing [93].

#### 3.2.3. Systemic Therapy for Metastatic RMS

Attempts at improving outcomes through the intensification of systemic therapy via the addition of active agents in metastatic RMS have been largely unsuccessful [94]. Although outcomes over time are marginally improved in comparison with historical cohorts, there have been no statistically significant improvements seen in EFS or OS that are attributable to the addition of new agents to the standard backbone regimen. The marginal improvements in outcome are likely to be multifactorial, for example, secondary to improved supportive care and improvements in local control techniques. Over the years and across paediatric oncology consortia, if a chemotherapeutic agent (or combination) was found to be active in preclinical RMS models, it has been tested in a window study. Therapeutic window studies have been conducted with the goal of confirming the utility of chemotherapeutics in high-risk patients.

Patients with metastatic disease and especially those with >one Oberlin risk factor remain a challenging population to treat. Therapy intensification has included adding active or targeted agents to the standard ifosfamide, vincristine, actinomycin D (IVA) or vincristine, actinomycin D, cyclophosphamide (VAC) backbone chemotherapies, dose escalation, the delivery of chemotherapy on an interval-compressed schedule, administering high-dose chemotherapy with stem cell rescue (SCR) and/or prolonging therapy with known active agents in the form of maintenance. Table 3 lists completed international clinical trials which have recruited patients with metastatic RMS over the last few decades. In the following sections we detail the conclusions drawn from various cooperative group studies over time that are relevant to the treatment of metastatic RMS.

##### Adding Active Agents to Standard Backbone

Despite numerous modifications to systemic chemotherapy regimens, outcomes for patients with metastatic RMS have not improved significantly over time. Early trials such as IRS I and II showed no improvement in outcome in patients with stage IV RMS following the addition of doxorubicin to a VAC backbone [95,96,113,114]. IRS III introduced cisplatin and etoposide to select patients, showing modest improvements in progression-free survival (PFS) and OS for certain primary tumour sites [97]. Subsequent IRS IV and other IRSG/COG studies (as shown in Table 3) identified active agents through phase II window trials, but integrating these into standard regimens failed to improve survival. Notably, the D9802 study demonstrated that vincristine–irinotecan (VIr) was highly active in newly diagnosed metastatic RMS [100]. The ARST0431 trial, which used an intensified multi-agent regimen including VIr, showed improved outcomes (3-year EFS 69%), but only in patients with favourable risk profiles (0–1 Oberlin risk factors) [88].

European trials like RMS79 and CWS-81/86 also did not yield major advances. RMS79 showed no benefit from different actinomycin-D schedules [104]. CWS-81 had dismal survival outcomes (11% 5-year DFS), while CWS-86 slightly improved outcomes (19% 5-year EFS) with a shortened VAIA regimen [109,110].

The MMT 98 study confirmed the safety but limited efficacy of carboplatin in RMS [107]. However, a doxorubicin window within MMT 98 showed a promising 60% partial response rate, reinforcing its activity in RMS, though without definitive survival benefit [107].

The E*p*SSG subsequently adopted doxorubicin combined with standard IVA chemotherapy (IVADo) the first four cycles of induction treatment to a total doxorubicin dose of 240 mg/m^2^ for metastatic RMS within the BERNIE and MTS 2008 studies. Patients in both studies also received 12 months of vinorelbine and cyclophosphamide maintenance chemotherapy as the standard of care (see Maintenance Chemotherapy Section). In a pooled analysis of patients from both studies, 372 RMS patients with a median follow-up of 55.2 months had a 3-year EFS and OS of 35.5% and 49.3%, respectively [6]. Patients with <two Oberlin risk factors had improved outcomes compared with patients with three or four risk factors (3-year EFS of 46.1% versus 12.5% and 3-year OS of 60.0% versus 26.0%) [6]. Patients with metastatic RMS treated as per CWS guidelines with CEVAIE during a similar era had companiable outcomes [11].

##### Adding Targeted Agents to Standard Backbone

Several recent trials were designed to incorporate targeted agents with standard backbone chemotherapy in frontline metastatic RMS. The BERNIE phase II study randomised patients with metastatic STS (*n* = 103 with RMS) to standard IVADo/IVA chemotherapy with or without bevacizumab, a monoclonal antibody targeting vascular endothelial growth factor (VEGF) [108]. Since STS significantly overexpresses angiogenic VEGF, the rationale was to inhibit tumour angiogenesis. The addition of bevacizumab to backbone chemotherapy was tolerable but there was no significant difference in median EFS between the STS patients who did or did not receive bevacizumab and the 2-year EFS was 41% for RMS patients within both groups. The study was unable to establish prognostic value for bevacizumab-related plasma or tumour biomarkers [108].

A COG study (ARST08P1) evaluated the feasibility and efficacy of adding cixutumumab (insulin-like growth factor-1 monoclonal antibody) or temozolomide to the ARST0431 intensive chemotherapy backbone (VDC/IE/VIr/VAC) in patients with metastatic RMS. No additive toxicities were noted but the addition of neither cixutumumab (3-year EFS 16%) nor temozolomide (3-year EFS 18%) showed a survival benefit [103].

##### Dose Intensification with Interval-Compressed Chemotherapy

The high-risk COG study ARST0431 for patients with metastatic disease trialled a multipronged approach to improve outcomes in this patient subset [88]. Not only were active agents (irinotecan, doxorubicin, etoposide and ifosfamide) in RMS added to VAC backbone chemotherapy but irinotecan also doubled as a radiosensitizer and VDC/IE chemotherapy was delivered according to an interval-compressed schedule with chemotherapy cycles being delivered every 14 days with GCSF support. All M1 patients included in ARST0431 (*n* = 109) were reported to have a 3-year EFS and OS of 38% and 56%, respectively [88]. This was improved compared with the preceding survival reported in the IRSG/COG window studies (best reported outcomes were with the combinations of ifosfamide/doxorubicin with a 3-year FFS of 25% and OS of 39%and ifosfamide/etoposide with a 3-year FFS of 33% and OS of 55%) and was very similar to the E*p*SSG’s MTS 2008 outcome [81,115]. Patients with 0–1 Oberlin risk factors had a 3-year EFS of 67% which was improved compared with historical cohorts and those with 2 Oberlin risk factors had a 3-year EFS of 19% [88]. One of the challenges of the design of ARST0431 was the inability to discern which of the three interventions (the use of irinotecan as a radiosensitizer; the use of interval-compressed cycles; or the use of multiple known active agents) provided the most patient benefit and the results were confounded by the finding that the FN < 10 year cohort (subsequently treated as IR in COG studies) accounted for the improved EFS and OS for the entire patient cohort. Dose intensification through the addition of irinotecan on days 8–12 of a standard 21-day IVA cycle (IrIVA) is currently being investigated in the FaR-RMS study [4].

##### High-Dose Chemotherapy with Stem Cell Rescue

Several clinical trials (MMT4 91, RMS 4.99 and MMT 98) have investigated the role of myeloablative chemotherapy with SCR in an effort to intensify systemic therapy for the highest risk patients with metastatic disease. There was no significant improvement in survival outcomes but an increased risk of treatment-related morbidity was found [105,107,112]. A Cochrane systematic review of high-dose chemotherapy for children and young adults with stage IV RMS concluded that the use of high-dose chemotherapy (HDC) with SCR as a standard therapy in metastatic RMS is not justified by the existing literature but that selection bias could have contributed to the poor outcome for patients with adverse risk factors who were treated with HDC and SCR, resulting in an underestimation of efficacy [116]. No randomised controlled trial to address the efficacy of HDC and SCR has been conducted to date in patients with RMS. The toxicity associated with HDC currently excludes this as a standard option in patients with metastatic RMS [116,117].

##### Maintenance Chemotherapy

A prolonged duration of treatment and metronomic dosing may both contribute to a maintenance-type approach in RMS [118]. Minimal residual disease may be present even when disease shows complete resolution on imaging. Although not typically thought of as maintenance chemotherapy, patients on IRS I and II had prolonged treatment periods for up to 2 years, during which time cyclophosphamide was given orally. CWS 96 allocated metastatic patients (by physician choice) into two groups following carboplatin, epirubicin, vincristine, actinomycin D, ifosfamide and etoposide (CEVAIE) to either receive high-dose chemotherapy with thiotepa, cyclophosphamide, melphalan and etoposide followed by autologous stem cell return or low-dose oral metronomic maintenance therapy with trofosfamide and idarubicin (TI) alternating with trofosfamide and etoposide (TE) [11,111]. Oral maintenance therapy appeared to show an improved outcome, but selection bias could not be eliminated. This was one of the first studies to pave the way towards the further exploration of this approach. A more recent analysis of patients from the CWS-IV2002 and DOKIV 2004 trials concluded that maintenance chemotherapy was superior to allogeneic haematopoietic stem cell transplant or high-dose chemotherapy in metastatic RMS. However, in addition to inherent selection bias in this non-randomised comparison, treatment arms were significantly imbalanced, bringing into question the strength of this evidence [119].

Vinorelbine and cyclophosphamide chemotherapy is an active combination both in the relapse setting and in the frontline setting in High-Risk (HR) and VHR patients [120,121]. Following the completion of 25 weeks of IVADo, patients in the BERNIE and E*p*SSG MTS 2008 studies received 12 months of oral maintenance chemotherapy with vinorelbine and cyclophosphamide in a non-randomised fashion. Outcomes in metastatic RMS were modestly improved compared with previous studies but the contribution of maintenance chemotherapy could not be clearly ascertained [6]. Of 270 patients, 125 (46%) developed progressive disease, had an insufficient response, relapsed or died either during/at the completion of induction (*n* = 65) or during maintenance treatment (*n* = 60) [6]. The FaR-RMS study is exploring prolonging maintenance treatment, with metastatic patients being randomised between 12 and 24 months of maintenance chemotherapy [4].

Outcomes for patients with metastatic disease remain suboptimal despite the intensification of systemic therapy via the addition of known active and targeted agents, the use of HDC and SCR and the addition of maintenance chemotherapy. New approaches are urgently required to address this ongoing unmet need.

#### 3.2.4. Local Control in Metastatic RMS

In the E*p*SSG protocols, disease reassessment occurs after six cycles of induction chemotherapy in patients with metastatic RMS, with an aim to initiate the delivery of local control in the form of surgery and/or RT from week 22 (i.e., following cycle seven) in most cases.

1. Surgical Resection

Primary surgical excision is rarely indicated in the setting of metastatic disease. The delayed resection of the primary site following neoadjuvant chemotherapy is the preferred approach, where appropriate. Surgery to metastatic sites is sometimes considered in individual cases, especially in patients with oligometastatic disease, though the mainstay of local therapy for metastatic sites is RT.

2. Radiotherapy

RT is recommended for the primary tumour site, any involved locoregional lymph nodes and as far as possible to all metastatic disease sites [4,6,122]. The timing for RT in metastatic disease is similar in the COG (week 20) protocols. The RT dose is dependent on the post induction chemotherapy tumour volume and for patients with multiple sites of metastatic disease, some sites may be prioritised, with RT to other sites being deferred. Dávila Fajardo et al. recently published an overview on the details and specificities of RT for RMS [122].

Despite the recommendation for all sites of disease to receive RT, data from MTS 2008 and BERNIE revealed that only 22% of all patients received RT to all metastatic sites. A landmark analysis of RMS patients from the BERNIE study reported better outcomes for patients receiving RT to some or all disease sites [123]. The analysis of MTS 2008 data confirmed that the 3-year PFS was impacted by the extent of RT and was highest in patients receiving irradiation to all disease sites (radical RT; 3-year PFS 62%), lower in those receiving partial RT (3-year PFS 39.5%) and lowest in patients receiving no RT (3-year PFS 30.1%) [123,124]. Ferrari and colleagues retrospectively reviewed a cohort of 80 paediatric and AYA patients with M1 RMS treated between 1990 and 2020 and found that patients who had RT to all metastatic sites had an improved 5-year EFS and OS of 70.6% and 76%, respectively [125]. These retrospective findings cautiously suggest that RT to all disease sites may influence the outcome but must be interpreted cautiously in view of the retrospective nature of the analyses and the risk of bias.

Although RT to all feasible metastatic sites is generally considered in the paediatric setting, this is not routinely the case in adults. Whether this influences the differential age-related outcome in RMS remains unclear. The delivery of RT in metastatic patients may have several attendant toxicities including myelosuppression, limiting the timely delivery of systemic therapy and increasing the susceptibility to infections. Furthermore, RT to multiple sites of disease can be a logistical challenge. In the case of oligometastatic disease, and when technically feasible, stereotactic ablative body radiotherapy (SABR) may be considered. Local treatment in the form of RT to all or some metastatic sites has been a topic of debate. The question of whether the benefit of complex, multi-site RT outweighs the burden of this intense treatment on these patients with a very poor prognosis is being prospectively addressed in a randomised study within the FaR-RMS trial [4,122].

3. Cytoreductive Surgery and Hyperthermic Intraperitoneal Chemotherapy

Peritoneal sarcomatosis can occur in children and young people with abdomino-pelvic RMS and presents a therapeutic challenge: an R1 resection is often not possible and limitations to whole abdomen RT exist due to the tolerance of the overlying bowel [126]. In this setting, CYP with RMS have been included in trials assessing the feasibility and safety of hyperthermic intraperitoneal chemotherapy (HIPEC) in specialised centres where the procedure has been well tolerated [127,128]. A recent review summarising reported cases in the literature included some apparent success in RMS but concluded that the impact of HIPEC versus systemic chemotherapy and debulking surgery on OS in paediatric malignancies is uncertain due to the lack of clinical trials and very small sample size across tumour subsets [129].

#### 3.2.5. Biologic Challenges in Metastatic RMS

An in-depth discussion of why metastatic disease has been challenging to treat for the last several decades is beyond the scope of this review, but it is indisputable that better treatment strategies specific for metastatic patients are urgently needed. *PAX3(7)::FOXO1* gene fusions encode potent aberrant transcription factors which impact growth, apoptosis and myogenic differentiation and stimulate metastasis [14,18]. Research on human primary RMS cell lines and animal models has shown that *PAX3(7)::FOXO1* expression levels at the cellular level are heterogeneous [130]. To what degree this heterogeneity exists among primary and metastatic sites would be interesting to further understand. Variable expression levels may enable FP RMS cells to exploit their host on multiple levels and the selective targeting of *PAX3(7)::FOXO1* high expressors may not prove to be curative. It may also be important to understand the pathways which influence fluctuating *PAX3(7)::FOXO1* expression to explore treatments which may overcome plasticity [130]. Thus, the as-yet unsuccessful attempts to target the *PAX3(7)::FOXO1* fusion gene may not be the anticipated holy grail.

An analysis of the integrin expression profiles of circulating plasma exosomes isolated from cancer patients may be able to predict sites of future metastasis in patients, which would revolutionise how we risk stratify metastatic patients and help develop inhibitors of metastasis before it has occurred in patients [131,132].

The tumour microenvironment (TME) including the extracellular matrix and vascular, stromal and immune cells may have an impact on the interplay between RMS cells and their metastatic fate. The influences of hypoxia, metabolites and cytokines on tumour cells and the TME is likely to influence not only the proliferative abilities of cells but also their metastatic potential [133]. For example, HIF activation is known to promote angiogenesis, metabolic reprogramming and stem cell-ness, which all have a combined impact on invasion and metastases [134,135].

Radio-resistant RMS cell lines studied in the lab are characteristic of an aggressive and pro-metastatic phenotype that can resist the harmful effects on the cell of reactive oxygen species, engage DNA damage repair pathways, counteract RT-induced cell cycle arrest and express cancer stem cell-ness [136]. Whether these features of radio resistance are intrinsic, acquired or a combination of both and the underlying mechanisms whereby this occurs will be pivotal in understanding the pathophysiology, especially for patients with in-field or field-edge local failure following RT. The problem of radio resistance also emphasises the importance of exploring potential radio-sensitising approaches.

Achieving objective radiologic responses is possible for the majority of patients with metastatic RMS, although in the MTS 2008 study only 103/259 patients completed both induction and maintenance chemotherapy with a median time to event of 11.6 months [6]. Despite the multimodality approaches and attempts to intensify treatments, there has been relatively little progress towards improving outcomes over the last four decades; the main ongoing challenge is achieving sustained response/remission. The development of liquid biopsies as a tool in RMS may enable the non-invasive detection of residual/relapsed disease and the genetic evolution of the tumour to facilitate future treatment strategies [137,138].

### 3.3. Relapsed RMS

Recurrent RMS remains very challenging to treat, with poor outcomes for most patients. In the E*p*SSG RMS 2005 study, 29% of all patients with M0 RMS had an event within 5 years, and in the MTS 2008 study 66% of patients with M1 RMS had an event within 3 years (median time to event of 11 months) [5,6]. The three-year OS following relapse is <20% underlining the ongoing need to develop novel approaches to treatment and ultimately more effective treatments for these patients [139,140].

#### 3.3.1. Relapse Risk

There is a distinction between the relapse or progression of disease which occurs following an initial response to chemotherapy and the rarer primary refractory disease in which patients progress through treatment without an initial period of response. Refractory disease carries a very poor prognosis and makes up ~10% of the patients included in the VIT0910 study [140]. The majority of relapses occur after either a complete or partial response and the poorest outcomes among this group are those with relapse or progression while on treatment [141]. For patients who have completed frontline therapy, earlier relapses (<18 months from diagnosis) are associated with inferior survival [142].

There are several factors used in clinical risk stratification in newly diagnosed patients which correlate with an increased risk of relapse. These include age > 10 years or <1 year, histology (alveolar now replaced by *PAX3(7)::FOXO1* fusion-positive status), site (unfavourable), IRS group (higher group), size (>5 cm) and nodal involvement (N1). In multiple studies, an alveolar histology has been identified both as a risk factor for recurrence (both localised and metastatic) but also as a harbinger of poor outcome following treatment for relapsed disease [7,19]. After metastatic status, fusion status is currently recognised as the most important factor associated with the outcome in patients with RMS and currently ongoing studies will prospectively study this [4]. Over time, the definitions of an unfavourable site have evolved as we continue to refine the prognostic relevance of this clinical feature. Currently within E*p*SSG, unfavourable sites of localised disease include extremities, head and neck parameningeal and “other” sites, and for metastatic disease, extremities and “other” sites [4].

#### 3.3.2. Patterns of Relapse

Table 4 depicts the timing and pattern of relapse published by various cooperative groups over the last five decades. A reduction in both relapse rate and OS rates over time are attributed to the improved delivery of systemic treatment, which includes advancements in supportive care, as well as an improved ability to deliver RT, particularly in initially non-metastatic disease. An analysis of 695 patients with IRS group II RMS with or without resected positive lymph nodes treated on the IRS I-IV studies showed that distant failure rate (14%) exceeded local (8%) and regional (4%) failure rates. Over time, between 1972 and 1997, the distant failure rate decreased particularly in ERMS [143].

The pattern of relapse in RMS may vary and may include local, locoregional, oligometastatic or widely metastatic disease alone or in combination. For patients with initially localised RMS, most (64–76%) relapses involve the primary site of disease and draining nodal basin(s) [146,147,148]. Of these, 81–87% are local relapses involving only the primary site of disease and the remaining relapses occur in the locoregional lymph nodes with or without the involvement of the primary site [148]. Certain groups such as infants with incompletely resected IRS group III tumours have been noted to have higher rates of local failure which is attributable to the avoidance of aggressive local control approaches [149,150,151,152,153]. Most patients who have a metastatic recurrence have multiple synchronous metastases [145].

#### 3.3.3. Systemic Therapy for Relapsed RMS

Successful efforts to bring new therapies to the clinic for relapsed RMS patients have been limited. There are few randomised trials in the relapsed setting and some of these trials have been designed to identify new agents for use in the frontline setting rather than to assess treatments specific to the relapse setting. Outcomes are limited by reduced efficacy, fewer opportunities for effective local therapy, heavy pretreatment and possibly acquired resistance mechanisms.

The COG conducted a trial comparing response rates of two schedules of irinotecan and vincristine in patients with relapsed or refractory RMS and reported outcomes from 89 assessable patients. One schedule was a total dose per cycle of 200 mg/m^2^ of irinotecan given on days 1–5 and 8–12 of a 21 day cycle (response rate, 26%; 1-year FFS 37%; 1-year OS 55%) and the second was 250 mg/m^2^ given on days 1–5 of a 21 day cycle (response rate, 37%; 1-year FFS 38%; 1-year OS 60%). The study concluded that as there was no difference in response rates between the two irinotecan schedules, the shorter, more convenient regimen would be taken forward [154].

A phase II COG study randomising patients at first relapse to vinorelbine and cyclophosphamide with either bevacizumab or temsirolimus reported a 6-month EFS of 69.1% for temsirolimus vs. 54.6% for bevacizumab and objective response rates of 47% and 28%, respectively. The study had a “pick the winner” design with no standard arm. The temsirolimus arm was superior to the bevacizumab arm and superior to historical controls [155]. Temsirolimus was taken forward into the ARST1431 frontline IR study but did not improve outcomes when combined with standard backbone chemotherapy (3-year EFS 64.8% in the VAC/VIr group vs. 66.8% in the VAC/VI plus temsirolimus group) [24].

Within E*p*SSG, a randomised phase II trial for relapsed (first or subsequent relapse) or refractory RMS patients tested the 5-day VI schedule with or without the addition of temozolomide and reported an overall response rate following two cycles of 44% for the vincristine, irinotecan and temozolomide (VIT) arm, which was superior to 31% for the VI arm [140]. Both PFS and OS were superior for the VIT arm (2-year OS 32% vs. 21%, *p* < 0.05; PFS 21% vs. 13%) in relapsed and refractory patients, although only OS was statistically significant [140]. VIT has been taken forward as the European standard of care in relapse for patients who received alkylating agents during first-line treatment. In the current FaR-RMS trial, relapsed RMS patients were randomised between VIT and VIr + regorafenib, a multi-tyrosine kinase inhibitor (TKI), following promising results with this novel combination in RMS in a phase Ib setting [4,156].

Patients have also been treated in non-randomised phase II studies. A recent COG publication described the outcome of 175 RMS patients treated in 13 COG single-agent phase II studies. Although not explicitly stated, this cohort was likely primarily comprised ofpatients who had had multiple relapses or whose disease was multi-refractory. In most cases, patients in first relapse are treated with multiple agents rather than single-agents. The six-month EFS was estimated at 16.8% and only trials of vinorelbine and rebeccamycin achieved their response-based primary endpoint [157].

Patients in the relapse setting who relapse/progress on more established phase II combinations are generally offered access to biomarker-driven phase I/II studies or targeted agents available through compassionate or extended use programmes based on the molecular profiling of the relapsed tumour. The COG MATCH trial (Molecular Analysis for Therapy choice; NCT 03155620) has included patients in phase II treatment arms of molecularly targeted therapies based on identified molecular variants in tumour samples [158]. A recent systematic review of early-phase studies that included children and young people with relapsed and refractory RMS found an objective response rate of only 22.6% and poor outcomes; but the study quality was limited by poor and inconsistent reporting [159].

Outside of the clinical trial setting, the choice of systemic and local therapy options may be made based on several considerations which have been recently outlined in an E*p*SSG summary of treatment recommendations in relapsed RMS [160].

#### 3.3.4. Local Control for Relapsed RMS

Several factors influence the decisions around local control at the time of relapse, at the forefront of which is curative vs. palliative intent, the location and extent of relapsed disease, whether or not a recurrence is in the field of previous RT and the organ/tissue tolerance in the region potentially requiring re-irradiation [139]. In the setting of the relapse of localised RMS, the absence of RT during primary treatment (for example in very young patients) not only makes this a favoured local control option where feasible but is also an independent positive prognostic indicator [142]. The current thinking on local control for relapsed RMS is described in the recently published E*p*SSG treatment recommendations for RMS [160].

#### 3.3.5. Outcomes of Relapsed RMS 

Several features of relapsed RMS may have an impact on survival outcome. In an analysis of localised (M0) patients from the European SIOP-MMT trials who had completed treatment, had CR/stable residual for at least 6 months from the end of treatment and at least 3 years of follow-up from the last event for alive patients, 37% of patients were alive at >3 years from their first relapse [142]. Outcomes following the relapse of metastatic RMS are extremely poor [82,161]. Patients whose disease recurs at distant metastatic sites have an abysmal prognosis with a reported 5-year post relapse OS of 0–10% [12,82,113,142,144,145]. A better prognostic group among relapsed patients includes those with local or locoregional recurrences [141]. Patients with a large tumour (>5 cm in maximal diameter) at relapse have also been identified as a poorer prognostic group [146]. The time to progression has been associated with time outcome in relapsed patients. Like several of the preceding features, this can also be described as a spectrum [139]. Patients whose disease recurs while on treatment or those whose disease progresses through treatment carry the worst prognosis (5-year OS 2–8%), while prognosis for those who relapse following a period of clinical remission varies according to the time to recurrence [157]. The four-year post relapse survival was 12%, 21%,and 41%, respectively, for those who relapsed <6 months, 6–12 months and >12 months, respectively, after the completion of initial therapy [162].

1. Relapse of Initially Localised RMS

Several groups have studied outcomes in patients who relapse after initial presentation with localised RMS [12,82,113,142,144,145]. Although in the early IRSG studies patients with both localised and metastatic disease were included in the analyses, given that the prognoses are so different, relapsed patients with initially metastatic disease should be separately analysed to determine the best prognostic features to risk stratify this patient group.

An analysis of pooled data from the Italian studies RMS 79, 88 and 96 identified alveolar subtype, parameningeal or other site, metastatic recurrence and on-treatment recurrence as independent risk factors for poor survival following relapse [144]. On the basis of this analysis, the authors developed a risk stratification model predicting post relapse 5-year OS which was 16% in patients with two risk factors, while no patients with three or more risk factors survived [144].

In the setting of initially localised RMS, tumour-related factors (metastatic relapse, large size, unfavourable site, nodal involvement at initial diagnosis, alveolar histology, time to relapse < 18 months) and treatment-related factors (prior RT and chemotherapy agents used) adversely influence the chance of a cure with second-line treatment [142]. An analysis completed by Chisholm et al. used a weighted scoring system to generate a nomogram, allowing an estimation of the chance of salvage following the first relapse of localised RMS [142].

The COG pooled data from IRS III-IV identified histologic subtype as strongly correlating with post relapse survival [12]. Among patients with alveolar RMS, the only good prognostic factor was favourable site (orbital, paratesticular or vaginal primary) while for patients with embryonal RMS, stage, pattern of relapse and chemotherapy received were found to have prognostic significance [12].

Pooled data from multiple CWS studies (CWS-81, CWS-86, CWS-91 and CWS-96) found age, histology, tumour size, tumour site, post surgical stage and the omission of RT to be factors associated with an increased risk or recurrence [145].

2. Relapse of Initially Metastatic RMS

Although few patients with metastatic RMS appear to be salvaged following relapse, a small proportion do survive. A three-year OS of 10% was reported by the CWS group in 129 patients with the relapse of metastatic RMS, and the COG reported 12% 5-year OS in relapsed metastatic embryonal RMS and 3% in patients with group II, III and IV alveolar RMS [12,161]. An analysis of pooled data within INSTRuCT looking at survival after first recurrence in initially metastatic patients has recently been completed (In press).. Randomised trials of relapsed RMS have historically included the relapse of both metastatic and localised disease: in the future, the further refinement of risk factors for patient stratification may be considered.

## 4. Looking Forward—Advances in Developmental Diagnostics and Therapeutics

### 4.1. Diagnostics

Diagnostic radiology is a constantly developing field with the implementation of techniques such as whole-body diffusion-weighted MRI with background suppression (DWIBS) and FDG-PET/MRI. In recent years several potential game changers have been introduced. First, there was the advent of photon counting computed tomography, a technique with a higher contrast-to-noise ratio, improved spatial resolution and optimised spectral imaging compared with conventional CT [163]. This can impact diagnostics due to a higher resolution, e.g., in head and neck RMS where osseous involvement may be detected earlier. Perhaps more exciting is the implementation of novel contrast agents and their potential use in molecular imaging [164]. A development in the field of nuclear medicine, Long Axial Field of View (LAFOV) PET-CT, makes it possible to image the whole body in a single field of view with higher resolution images, decreased radiation exposure and shorter scan times [165,166]. In a study of 10 children with neuroblastoma, scan times were as short as 10 min and none of the children required sedation for the scan [167]. Finally, the introduction of ^68^Ga-FAPI PET/CT is based on evidence that the stroma of most sarcomas overexpresses Fibroblast Activation Protein (FAP), a type II transmembrane serine protease [168,169]. Using FAP Inhibitors (FAPIs) as a radiotracer may lead to a higher diagnostic sensitivity of PET imaging. Preliminary studies with a small number of patients have shown that in a head-to-head comparison ^68^Ga-FAPI PET/CT outperformed 18FDG PET/CT in adult patients [170]. If indeed it is as selective as it seems to be, this new tracer could also be used for FAP-targeted radioligand therapy in the future. The classic radiologic size response to neoadjuvant chemotherapy has not shown to be prognostic for outcome [171,172]. Currently there is no early surrogate outcome measure in RMS. Therefore, in the FaR-RMS study, diffusion-weighted MRI response (both in the primary and relapsed setting) and ^18^FDG-PET/CT response (in the primary setting) are prospectively being investigated as potential early surrogate outcome measures [4].

The potential of new diagnostic techniques, some of which are still experimental, must be evaluated in prospective studies, where important questions around the potential of the upstaging of patient, and the subsequent risk of overtreatment, should be explored.

### 4.2. Minimally Invasive Treatments/Interventional Oncology

In the adult oncology setting, the minimally invasive treatment of metastatic disease using techniques such as trans-arterial chemoembolization (TACE), thermal ablation, cryotherapy and irreversible electroporation (IRE) are widely used in the palliative phase of care [173,174,175]. The recent advances in interventional oncology, a sub-discipline of interventional radiology, necessitate its inclusion in multimodality treatment and has been coined the fourth pillar of oncology [176]. Even though there have been early adopters of some of these approaches, the general uptake of therapeutic interventions in paediatric oncology has been relatively slow [177,178,179]. The implementation of interventional radiological techniques within RMS treatment will require collaboration with paediatric interventional radiologists and the inclusion of new techniques in prospective clinical trials like FaR-RMS.

### 4.3. Detecting Relapse: Current and Future Paradigms

Routine surveillance with a combination of the cross-sectional imaging of the primary site and/or metastatic sites and chest x-ray is recommended for up to 5 years following the completion of treatment, with most frequent surveillance in the initial two years after treatment. A comprehensive European imaging guideline for RMS has recently been published [171,180]. A study of surveillance imaging in localised RMS showed that the majority (61%) of relapses were detected at a time of clinical symptoms and there was no survival advantage for patients whose asymptomatic relapse was detected by routine surveillance [147]. At present, there is no role for FDG-PET imaging in the setting of routine surveillance imaging and it is only performed as part of re-staging once relapse has been confirmed.

Cell-free DNA (cfDNA), small DNA fragments derived from dead cells found in the blood and other body fluids, may arise from tumour shedding and have potential future utility as biomarkers in solid tumour patients. A surrogate for repeat tumour biopsies, these liquid biopsies allow for the detection of *PAX3(7)::FOXO1* in FP cases and other variants of interest which can be detected in FN RMS. The use of minimally invasive techniques to detect recurrence earlier (routine blood sampling to assess for circulating tumour DNA (ctDNA) or Circulating Tumour Cells (CTCs) for example) is currently under investigation [181,182]. ctDNA levels correlate with the clinical and radiologic response in pilot studies and will be investigated further in a cohort within the FaR-RMS study [4]. If this approach proves feasible and laboratory assays can be harmonised, it holds the possibility of the earlier detection of relapsed disease and may identify patients with the most potential for benefit from enrolment in early-phase trials at an earlier time in the patient journey. The prospective collection of ctDNA for the purposes of monitoring treatment response, assessing utility as an earlier biomarker of recurrence than conventional imaging, and providing information about the genetic makeup of a tumour and how this evolves over time may have implications on how disease monitoring occurs in the future [181,183,184,185,186]. The prognostic value of liquid biopsies in an IR RMS study has recently been published by the COG and both patients with FN and FP RMS with detectable ctDNA at diagnosis had poorer outcomes than those without [138].

### 4.4. Novel Therapies

As more genomic and molecular data from patients with RMS becomes available, particularly from patients enrolled on trials, we will be able to couple this data with patient outcomes and develop a more in-depth understanding of biologic subtypes. Re-biopsy at the time of relapse can potentially provide information on novel genomic alterations and tumour evolution. There is also great potential in the use of liquid biopsies to monitor disease burden and identify molecular biomarkers of predictive or prognostic value. The timely identification of therapeutic targets is not only important to determine eligibility for ongoing early-phase trials but also has the promise of providing insight into disease resistance mechanisms that must be better understood as we aim for improved patient outcomes in relapsed patients. Tumour-agnostic trials investigating the targeting of *ALK*, *HDAC*, *RAS/PI3K*, *FGFR4*, *CXCR4*, *IGFR1*, *MET* and *PDGFRA* have so far not given rise to data that would currently support the use of a targeted agent as monotherapy at second or subsequent relapse so combined testing with traditional systemic chemotherapy is needed in future studies as preclinical work to support this continues [187,188]. Table 5 lists completed phase I and II trials in which patients with relapsed RMS were eligible. 

### 4.5. Immune Checkpoint Inhibition

Immunotherapy in the form of immune checkpoint inhibition (ICI) has thus far not shown impressive responses in paediatric embryonal tumours. Whilst the reasons for this are incompletely understood, several factors probably contribute. TME in paediatric sarcomas is highly immunosuppressive, attributed to regulatory T cells and myeloid-derived suppressor cells, and this may curtail the efficacy of ICI [189]. The lack of a high mutational burden in many paediatric and TYA cancers, which is particularly relevant to FP RMS, suggests many of these patients lack natural adaptive immunity. It remains to be seen however if there is a differential response between FP and FN RMS (typically with a relatively higher mutational burden than FP RMS) when treated with ICI. Finally, it is possible that a more incompletely developed immune system in younger children may play a contributing role.

### 4.6. Engineered T Cell Therapy

Due to the lack of an endogenous immune response to a tumour, an alternate strategy is engineering patient T cells to be redirected to the cancer. This can be in the form of viral transductions with a transgenic T cell receptor or with a chimeric antigen receptor to develop chimeric antigen receptor-T (CAR-T) cells. Tumour antigens may be products of mutated genes, over expressed or aberrantly expressed cellular proteins, altered cell surface glycolipids or glycoproteins and cell-type-specific differentiation antigens [190]. These antigens may be present on both tumour cells as well as normal cells, albeit to a lesser extent, causing “on-target off-tumour toxicity” when treated with CAR-T cells. A biochemical and metabolic milieu unfavourable to T cell effector function combined with immunosuppressive signals from cancer and stromal cells are important factors that likely impact engineered T cell efficacy for solid tumours such as RMS. T cell activation without exhaustion, expansion, persistence and memory cell development has the potential to provide long-lasting defence against recurrent disease. Although there have been several well described barriers to the use of CAR-T therapy in solid tumours, the engraftment of CAR-T cells can be improved via the use of lymphodepleting conditioning regimens and improvements in CAR-T cell persistence are being investigated through additional engineering to enhance persistence [191].

There are several currently ongoing clinical trials with CAR-T cell therapy in solid tumours in children and young adults and although neuroblastoma and CNS tumours are leading in this frontier, CAR-T cells targeting *HER2*, *GD2*, *B7H3*, *PSMA*, *CD171*, *GPC3*, *IL13Ra2*, *EGFR806*, *CD55V6* and *CD276* have been trialled in children and young people and future targets in RMS may include *FGFR4*, *SLC19A1*, *EPHB4* and *ACVR2A*. The knowledge of target antigens, co-stimulatory molecules, safety mechanisms and safety profiles will be important in understanding the utility of CAR-T cells for relapsed patients with RMS.

### 4.7. Antibody Drug Conjugates

Antibody drug conjugates (ADCs) are a class of therapeutics, which although administered systemically at a high concentration of drug, have their efficacy enhanced and toxicity diminished through the targeting of highly expressed tumour specific antigens which allow a specific antibody to deliver a covalently linked drug payload [192]. Many of the targets identified for CAR-T development are also attractive ADC candidates for RMS. As this class of agents expands, the aim of ADC delivery to tumour/metastatic sites in a selective fashion is paramount and holds the promise of an improved therapeutic index [192]. Factors which will need to be overcome in the setting of relapsed sarcomas in order for ADCs to advance in the clinic include the identification of tumour-specific target antigens and the optimisation of delivery to heterogenous tumours impacted by a myriad of variables within the TME.

### 4.8. Therapeutic Cancer Vaccines

Therapeutic cancer vaccines have thus far failed to show benefits in most paediatric malignancies [193] and RMS is no exception, specifically with attempts to therapeutically exploit the *PAX3(7)::FOXO1* breakpoint region., Further attempts in this field are impeded by limited HLA class I or class II antigen–peptide binding [194]. The currently ongoing PerVision study (NCT06094101) is a multicentre, open-label, phase I/II feasibility and early proof of concept study open to the recruitment of patients with metastatic sarcomas that have a fusion. The trial enables the delivery of a bespoke sarcoma-specific individualised cancer peptide vaccine to patients.

## 5. Conclusions

We have described the clinical and biological features of the poorest risk subgroups in patients with RMS and summarised the attempts to date to improve outcomes. Further research is needed to identify new robust biomarkers that can help define the poorest risk RMS subgroups and to progress our understanding of factors contributing to aggressive biology. It will be essential to explore how to pharmacologically and immunologically exploit vulnerabilities, validate predictive models of relapse risk and develop mechanisms to counter resistance. Ultimately there is a need to make significant advances in systemic treatments for these patients while simultaneously continuing to improve local therapies.

## Figures and Tables

**Table 1:** E*p*SSG current risk stratification in RMS.

Risk Group	Subgroup	OS (%) *	EFS (%) *	Fusion	IRS Group	Site	Nodes	Size or Age
Low	A	96.7	93.7	FN	I	Any	N0	Both Fav
Standard	B	93.2	87.4	FN	I	Any	N0	One/both Unf
	C	93.4	76.9	FN	II, III	Fav	N0	Any
High	D	84.0	73.3	FN	II, III	Unf	N0	Any
	E	76.7	67.3	FN	II, III	Any	N1	Any
	F			FP	I, II, III	Any	N0	Any
Very High	G	49.7	48.8	FP	II, III	Any	N1	Any
	H	49.3 **	35.5 **	Any	IV	Any	Any	Any

FN: fusion negative; FP: fusion positive; N0: no locoregional nodal involvement; N1: locoregional nodal involvement; Fav: favourable; Unf: unfavourable; favourable sites include orbital, non-parameningeal head and neck, genito-urinary including bladder and prostate and biliary primaries; unfavourable sites include all other sites; favourable age: >1 yr and <10 yrs; favourable size: ≤5 cm in maximal diameter; unfavourable size: >5 cm in maximal diameter; IRS group I: localised tumour, completely removed with pathologically clear margins and no regional lymph node involvement; IRS group II: localised tumour, grossly removed with (a) microscopically involved margins, (b) involved, grossly resected regional lymph nodes, (c) both; IRS group III: localised tumour, with gross residual disease afterincomplete resection, or biopsy only; IRS group IV: distant metastases present at diagnosis; * 5-year EFS and OS Based on RMS-2005 Data [5]; ** 3-year EFS and OS [6].

**Table 2:** Modalities to detect metastatic disease in staging of rhabdomyosarcoma.

Site	Modality	Comment
Primary site	MRI	Imaging should include regional LN.
Lungs	CT	Definition of lung metastases: ≥1 nodule(s) ≥10 mm max diameter; ≥2 nodules 5–10 mm diameter; or ≥5 nodules <5 mm diameter. Lesions not fulfilling these criteria should be regarded as indeterminate nodules, do not require biopsy and will not upstage to Very High Risk.
Bones	^18^FDG PET-CT/MR	
Distant or regional LN	^18^FDG PET-CT/MR, WB-MRI	Any LNs beyond regional nodal basin are considered distant. If suspicion, sampling is indicated. If radiographically normal but primary is paratesticular or extremity, sampling is recommended.
Bone marrow	^18^FDG PET-CT/MR, WBMRI, BMA/Bx	Variations in practice for BMA/Bx; false negative ^18^FDG PET-CT is rare.
Pleural/peritoneal nodules	MRI or CT	If present, consider M1.
Serosal effusions	MRI	If no nodularity, but moderate- to high-volume effusion, cytologic examination recommended to look for malignant cells.
CSF	CSF cytology	Part of staging in parameningeal RMS. If positive, consider M1.
Multifocal disease	MRI or CT	If present, consider M1.

MRI: magnetic resonance imaging; CT: computed tomography; FDG PET: ^18^fluorodeoxyglucose positron emission tomography; WBMRI: whole-body MRI; LNs: lymph nodes; BM: bone marrow; BMA/Bx: bone marrow aspirate and biopsies; M1: metastatic; CSF: cerebrospinal fluid.

**Table 3:** Completed international clinical trials which recruited patients with metastatic rhabdomyosarcoma.

Consortium	Study	Years	Chemo	Author [Ref.]
IRSG	IRS I	1972–1978	VAC + Doxorubicin	Maurer [95]
IRSG	IRS II	1978–1984	VAC + Doxorubicin	Maurer [96]
IRSG	IRS III	1984–1991	VAC + Doxorubicin + Cisplat ± E	Crist [97]
IRSG	IRS IV	1991–1995	IE or Vincristine + Melphalan	Breneman [81]
IRSG	CCG6941/POG9490	1994–1996	Topo	Pappo [98]
IRSG	D9501	1996–1999	Window Topo/Cyclo	Walterhouse [99]
IRSG	D9802	1999–2000	Window Irinotecan ± vincristine	Pappo [100]
IRSG	D9803	1999–2005	VAC/VTC	Arndt [101]
COG	ARST0431	2006–2008	VDC/IE VIr/VAC	Weigel [88]
COG	ARST0531	2006–2012	VAC vs. VAC/VIr	Casey [102]
COG	ARST08P1	2010–2013	ARST0431 backbone + cixu or TMZ	Malempati [103]
COG	ARST1431	2016–2022	VAC/VIr vs. VAC/VIr +Temsirolimus	Gupta [24]
ICG	RMS79	1979–1987	VDC/VAC (2 schedules Actinomycin)	Carli [104]
MMT	MMT89	1989–1991	CEV/IVA/VIE	Carli [105]
MMT	MMT91	1991–1995	CEV/IVA/VIE/Mephalan + ASCR	Carli [105]
MMT	MMT95	1995–2003	IVA/IVA + carbo, epirubicin, E	Oberlin [106]
MMT	MMT98	1998–2005	6-drug + VAC maintenance × 9 cycles	McDowell [107]
E*p*SSG	MTS2008	2010–2016	IVADO	Schoot [6]
E*p*SSG	BERNIE	2010–2016	IVADO ± Bevacizumab	Chisholm [108]
CWS	CWS81	1981–1986	VACA	Koscielniak [109]
CWS	CWS86	1985–1990	VAIA	Koscielniak [110]
CWS	CWS91	1990–1995	EVAIA or CEV/IVA/VIE + HDC	Sparber-Sauer [92]
CWS	CWS96	1995–2003	CEVAIE + HD or OMT	Klingebiel [111]
CWS	CWS-IV 2002	2002–2010	Topo/Carbo + IVA/IVDo + Topo/Cyclo or IVA/CEV/IVE	Heinz [11]
CWS	CWS DOK IV 2004	2002–2010	CEVAIE or VAIA	Heinz [11]
AIEOP	RMS 4.99	1999–2009	HDC	Bisogno [112]

IRSG: Intergroup Rhabdomyosarcoma Study Group; COG: Children’s Oncology Group; ICG: Italian Cooperative Group; MMT: Malignant Mesenchymal Tumour Group; E*p*SSG: European paediatric Soft tissue Sarcoma Group; CWS: Cooperative Weichteilsarkom Studiengruppe; AIEOP: Italian Association of Pediatric Hematology and Oncology; VAC: vincristine, actinomycin, cyclophosphamide; E: etoposide; IE: ifosfamide/etoposide; Cisplat: cisplatin; Cyclo: cyclophosphamide; Carbo: carboplatin; Topo: topotecan; VTC: vincristine, topotecan, cyclophosphamide; VDC: vincristine, doxorubicin, cyclophosphamide; VIr: vincristine, irinotecan; Cixu: Cixutumumab; TMZ: temozolomide; CEV: carboplatin, etoposide, vincristine; IVA: ifosfamide, vincristine, actinomycin; VIE: vincristine, ifosfamide, epirubicin; ASCR: autologous stem cell rescue; IVADO: ifosfamide, vincristine, actinomycin, doxorubicin; VACA: vincristine, Adriamycin, cyclophosphamide, actinomycin; VAIA: vincristine, Adriamycin, ifosfamide, actinomycin; EVAIA: etoposide, vincristine, adriamycin, ifosfamide, actinomycin; HDC: high-dose chemotherapy; CEVAIE: carboplatin, epirubicin, vincristine, actinomycin, ifosfamide, etoposide; OMT: oral metronomic therapy.

**Table 4:** Timing and patterns of relapse in RMS.

Reference	Raney [113]	Pappo [12]	Mazzoleni [144]	Dantonello [145]	Oberlin [82]	Chisholm [142]
**Consortium**	COG	COG	AIEOP	CWS	COG, MMT, AIEOP	MMT
**Studies**	IRS I	IRS III, IV pilot, IV	RMS 79, 88, 96	CWS 81, 86, 91, 96	IRS III, IV pilot, IV, MMT 84, 89, 91, 98, AIEOP RMS 4.99	MMT 84, 89, 95
**N**	115	605	125	337	788	474
**Diagnosis type**	M1 and M0	M1 and M0	M0	M0	M1	M0
**Relapse rate**	34%	26%	31%	29%	-	36%
**OS**	6% (5-year)	17% (5-year)	28.3% (5-year)	24% (5-year)	34% (3-year)	37% (3-year)
**MTTR (months)**	-	13.2	17.8	17.2	-	14
**Relapse Type**	M0 38% M1 62%	M0 46% M1 41% Unk 8%	M0 72% M1 28%	M0 64.4% M1 35.5%	-	M0 76% M1 24%

MTTR: median time to relapse; M0: non-metastatic; M1: metastatic; Unk: unknown; OS: overall survival; COG: Children’s Oncology Group; CWS: Cooperative Weichteilsarkom Studiengruppe; MMT: Malignant Mesenchymal Tumour Group; AIEOP: Italian Association of Pediatric Hematology and Oncology.

**Table 5:** Completed early-phase clinical trials in patients with relapsed solid tumours including RMS.

Agent	Class	Trial Identifier	Phase
Lorvotuzumab	ADC	NCT02452554	2
Tumour-Associated Antigen-Specific Cytotoxic T-Lymphocytes	Cellular therapy	NCT02239861	1
Eribulin mesylate + Irinotecan	Cytotoxic	NCT03245450	1 + 2
Nab-paclitaxel	Cytotoxic	NCT01962103	1 + 2
Vincristine/Irinotecan ± Temozolomide	Cytotoxic	NCT01355445	2
Trabectedin	Cytotoxic	NCT01453283	1
Temozolomide + O6-benzylguanine	Cytotoxic	NCT00020150	1
Liposomal doxorubicin	Cytotoxic	NCT00019630	1
Oxaliplatin + Irinotecan	Cytotoxic	NCT00101270	1
ABT-751	Cytotoxic	NCT00036959	1
Ixabepilone	Cytotoxic	NCT00030108	1
Vincristine + irinotecan	Cytotoxic	NCT00025363	2
Eribulin	Cytotoxic	NCT03441360	2
Exatecan	Cytotoxic	NCT00055939	2
Trabectedin	Cytotoxic	NCT00070109	2
Ixabepilone	Cytotoxic	NCT00331643	2
Pemetrexed	Cytotoxic	NCT00520936	2
Topotecan	Cytotoxic	NCT00003745	2
Irinotecan	Cytotoxic	NCT00004078	2
Auristatin	Cytotoxic	NCT00064220	2
Vinorelbine	Cytotoxic	NCT00003234	2
Arsenic trioxide	Cytotoxic	NCT00024258	2
Brostallicin	Cytotoxic	NCT00041249	2
Exatecan	Cytotoxic	NCT00041236	2
Docetaxel	Cytotoxic	NCT00002825	2
Oxaliplatin	Cytotoxic	NCT00091182	2
Becatecarin	Cytotoxic	NCT00006102	2
Sirolimus + celecoxib + etoposide/cyclophosphamide	Cytotoxic + mTORi	NCT01331135	1
Temsirolimus + liposomal doxorubicin	Cytotoxic + mTORi	NCT00949325	1 + 2
Vinblastine + Cyclophosphamide + Temsirolimus or Bevacizumab	Cytotoxic + mTORi	NCT01222715	2
Gefitinib + Irinotecan	Cytotoxic + TKI	NCT00132158	1
Surufatinib + Gemcitabine	Cytotoxic + TKI	NCT05093322	1 + 2
Sorafenib + Irinotecan	Cytotoxic + TKI	NCT01518413	1
Erlotinib + Temozolomide	Cytotoxic + TKI	NCT00077454	1
Continuous Hyperthermic Peritoneal Perfusion (Cisplatin)	HIPEC	NCT00436657	1
Bempegaldesleukin + Nivolumab	Immunotherapy	NCT04730349	1 + 2
Atezolizumab	Immunotherapy	NCT02541604	1
Tumour Vaccination + R-hIL-7 after cytotoxic chemo	Immunotherapy	NCT00923351	1 + 2
Nivolumab ± Ipilimumab	Immunotherapy	NCT02304458	1 + 2
Donor lymphocyte infusions	Immunotherapy	NCT00161187	1
JX-594 (Vaccinia GM-CSF/Thymidine Kinase-Deactivated Virus)	Intratumoural Local control	NCT01169584	1
R1507	MAb	NCT00642941	2
TB-403	MAb	NCT02748135	1
Enoblitzumab	MAb	NCT02982941	1
Cixutumumab + Doxorubicin	MAb	NCT00720174	1
Cixutumumab + Temsirolimus	MAb	NCT01614795	2
Cixutumumab	MAb	NCT00831844	2
Sonidegib	Other	NCT01125800	1 + 2
Afatinib	Other	NCT02372006	1 + 2
Vorinostat	Other	NCT00918489	2
Auto SCT followed by Cyclophosphamide + Thalidomide	Other	NCT01661400	1
Gallium nitrate	Other	NCT00002543	1
Simvastatin + Topotecan + Cyclophosphamide	Other	NCT02390843	1
Tanespimycin	Other	NCT00093821	1
Abemaciclib	Other	NCT02644460	1
Adavosertib + Irinotecan	Other	NCT02095132	1 + 2
Alvocidib	Other	NCT00012181	1
Talabostat + Temozolomide or Carboplatin	Other	NCT00303940	1
Alisertib	Other	NCT01154816	2
Temsirolimus	TKI	NCT00106353	1
Regorafenib	TKI	2013-003579-36	1
Cobimetinib	TKI	NCT02639546	1 + 2
Pazopanib	TKI	NCT01956669	2
Everolimus	TKI	NCT00187174	1
Lenvatinib + Everolimus	TKI	NCT03245151	1 + 2
Ceritinib	TKI	NCT01742286	1
Imatinib	TKI	NCT00006357	1 + 2
Regorafenib	TKI	NCT02048371	2
Sorafenib	TKI	NCT01502410	2
Dasatinib	TKI	NCT00464620	2
Crizotinib	TKI	NCT01524926	2
Imatinib	TKI	NCT00031915	2
Imatinib	TKI	NCT00154388	2
Decitabine + Vaccine Therapy	Vaccine	NCT01241162	1
Seneca Valley Virus-001 + Cyclophosphamide	Vaccine	NCT01048892	1
Herpes Simplex Virus-1 Mutant HSV1716	Vaccine	NCT00931931	1
Ganitumab + Dasatinib	Mab + TKI	NCT3041701	1
Vinorelbine + Mocetinostat	Cytotoxic + HDACi	NCT4299113	1
Prexasertib + Irinotecan	Cytotoxic + CHK1i	NCT4095221	1 + 2
Abemaciclib + Irinotecan or Irinotecan/Temozolomide	Cytotoxic + CDK4/6	NCT4238819	1
Sirolimus + metronomic chemotherapy	Cytotoxic + mTORi	NCT2574728	2

## Abbreviations

The following abbreviations are used in this manuscript:

ADC	antibody drug conjugate
AEIOP	Italian Association of Pediatric Hematology and Oncology
ARMS	alveolar rhabdomyosarcoma
ASCR	autologous stem cell rescue
AYA	adolescent and young adult
BMA/Bx	bone marrow aspirates and biopsies
CAR	chimeric antigen receptor
CEV	carboplatin, etoposide, vincristine
CEVAIE	carboplatin, epirubicin, vincristine, actinomycin D, ifosfamide, etoposide
cfDNA	cell-free DNA
Cisplat	cisplatin
Cixu	cixutumumab
COG	Children’s Oncology Group
CSF	cerebrospinal fluid
CT	computed tomography
CTCs	circulating tumour cells
ctDNA	circulating tumour DNA
CWS	Cooperative Weichteilsarkom Studiengruppe
Cyclo	cyclophosphamide
CYP	children and young people
DFS	disease-free survival
DWIBS	diffusion-weighted imaging with background suppression
E	etoposide
EFS	event-free survival
E*p*SSG	European Paediatric Soft Tissue Sarcoma Study Group
ERMS	embryonal rhabdomyosarcoma
EVAIA	etoposide, vincristine, adriamycin, ifosfamide, actinomycin
FAP	fibroblast activation protein
FAPI	fibroblast activation protein inhibitor
FaR-RMS	Frontline and Relapsed Rhabdomyosarcoma trial
FDG-PET	fluorodeoxyglucose positron emission tomography
FFS	failure free survival
FN	fusion negative
FP	fusion positive
HDC	high-dose chemotherapy
HIPEC	hyperthermic intraperitoneal chemotherapy
HR	high risk
ICG	Italian Cooperative group
ICI	immune checkpoint inhibitor
IE	ifosfamide, etoposide
INSTRuCT	International Soft Tissue Sarcoma Consortium
IR	intermediate risk
IRE	irreversible electroporation
IrIVA	irinotecan, ifosfamide, vincristine, actinomycin D
IRSG	Intergroup Rhabdomyosarcoma Study Group
IVA	ifosfamide, vincristine, actinomycin D
IVADo	ifosfamide, vincristine, actinomycin D, doxorubicin
LAFOV	long axial field of view
LFS	Li–Fraumeni Syndrome
LN(s)	lymph node(s)
LoH	loss of heterozygosity
LR	low risk
M0	non-metastatic
M1	metastatic
MMT	Malignant Mesenchymal Tumour group
MRI	magnetic resonance imaging
MTTR	median time to relapse
N0	no locoregional nodal disease
N1	locoregional nodal disease
OMT	oral metronomic therapy
OS	overall survival
PFS	progression-free survival
RMS	rhabdomyosarcoma
RT	radiotherapy
SABR	stereotactic ablative body radiotherapy
SCR	stem cell rescue
SIOP	International society of Pediatric Oncology
ssRMS	spindle cell/sclerosing rhabdomyosarcoma
STS	soft tissue sarcoma
TACE	trans-arterial chemoembolisation
TE	trophosfamide, etoposide
TI	trophosfamide, idarubicin
TKI	tyrosine kinase inhibitor
TME	tumour microenvironment
TMZ	temozolomide
Unk	unknown
VAC	vincristine, actinomycin D, cyclophosphamide
VACA	vincristine, actinomycin D, cyclophosphamide, adriamycin
VAIA	vincristine, actinomycin D, ifosfamide, adriamycin
VDC	vincristine, doxorubicin, cyclophosphamide
VEGF	vascular endothelial growth factor
VHR	Very High Risk
VIE	vincristine, ifosfamide, epirubicin
VIr	vincristine, irinotecan
VIT	vincristine, irinotecan, temozolomide
VLR	Very Low Risk
VTC	vincristine, topotecan, cyclophosphamide
WBMRI	whole-body magnetic resonance imaging
WHO	World Health Organisation
ZF	zinc finger

## References

[1] Skapek S.X., Ferrari A., Gupta A.A., Lupo P.J., Butler E., Shipley J., Barr F.G., Hawkins D.S. (2019). Rhabdomyosarcoma. Nat. Rev. Dis. Prim..

[2] Howlader N., Noone A.M., Krapcho M., Miller D., Brest A., Yu M., Ruhl J., Tatalovich Z., Mariotto A., Lewis D.R. (2021). SEER Cancer Statistics Review, 1975–2018.

[3] Siegel R.L., Miller K.D., Fuchs H.E., Jemal A. (2021). Cancer Statistics, 2021. CA Cancer J. Clin..

[4] Chisholm J., Mandeville H., Adams M., Minard-Collin V., Rogers T., Kelsey A., Shipley J., Van Rijn R.R., De Vries I., Van Ewijk R. (2024). Frontline and Relapsed Rhabdomyosarcoma (FAR-RMS) Clinical Trial: A Report from the European Paediatric Soft Tissue Sarcoma Study Group (EpSSG). Cancers.

[5] Bisogno G., Minard-Colin V., Zanetti I., Ferrari A., Gallego S., Dávila Fajardo R., Mandeville H., Kelsey A., Alaggio R., Orbach D. (2023). Nonmetastatic Rhabdomyosarcoma in Children and Adolescents: Overall Results of the European Pediatric Soft Tissue Sarcoma Study Group RMS2005 Study. J. Clin. Oncol..

[6] Schoot R.A., Chisholm J.C., Casanova M., Minard-Colin V., Geoerger B., Cameron A.L., Coppadoro B., Zanetti I., Orbach D., Kelsey A. (2022). Metastatic Rhabdomyosarcoma: Results of the European *Paediatric* Soft Tissue Sarcoma Study Group MTS 2008 Study and Pooled Analysis With the Concurrent BERNIE Study. J. Clin. Oncol..

[7] Gallego S., Zanetti I., Orbach D., Ranchère D., Shipley J., Zin A., Bergeron C., de Salvo G.L., Chisholm J., Ferrari A. (2018). Fusion status in patients with lymph node-positive (N1) alveolar rhabdomyosarcoma is a powerful predictor of prognosis: Experience of the European Paediatric Soft Tissue Sarcoma Study Group (EpSSG). Cancer.

[8] Di Carlo D., Chisholm J., Kelsey A., Alaggio R., Bisogno G., Minard-Colin V., Jenney M., Dávila Fajardo R., Merks J.H., Shipley J.M. (2023). Biological Role and Clinical Implications of MYOD1L122R Mutation in Rhabdomyosarcoma. Cancers.

[9] Le Loarer F., Cleven A.H., Bouvier C., Castex M.P., Romagosa C., Moreau A., Salas S., Bonhomme B., Gomez-Brouchet A., Laurent C. (2020). A subset of epithelioid and spindle cell rhabdomyosarcomas is associated with TFCP2 fusions and common ALK upregulation. Mod. Pathol..

[10] Shern J.F., Selfe J., Izquierdo E., Patidar R., Chou H.C., Song Y.K., Yohe M.E., Sindiri S., Wei J., Wen X. (2021). Genomic Classification and Clinical Outcome in Rhabdomyosarcoma: A Report From an International Consortium. J. Clin. Oncol..

[11] Heinz A.T., Schönstein A., Ebinger M., Fuchs J., Timmermann B., Seitz G., Vokuhl C., Münter M., Pajtler K.W., Stegmaier S. (2024). Cooperative Weichteilsarkom Studiengruppe. Significance of fusion status, Oberlin risk factors, local and maintenance treatment in pediatric and adolescent patients with metastatic rhabdomyosarcoma: Data of the European Soft Tissue Sarcoma Registry SoTiSaR. Pediatr. Blood Cancer.

[12] Pappo A.S., Anderson J.R., Crist W.M., Wharam M.D., Breitfeld P.P., Hawkins D., Raney R.B., Womer R.B., Parham D.M., Qualman S.J. (1999). Survival After Relapse in Children and Adolescents With Rhabdomyosarcoma: A Report From the Intergroup Rhabdomyosarcoma Study Group. J. Clin. Oncol..

[13] Dehner C.A., Rudzinski E.R., Davis J.L. (2024). Rhabdomyosarcoma: Updates on classification and the necessity of molecular testing beyond immunohistochemistry. Hum. Pathol..

[14] Parham D.M., Barr F.G. (2013). Classification of Rhabdomyosarcoma and its molecular basis. Adv. Anat. Pathol..

[15] Shern J.F., Chen L., Chmielecki J., Wei J.S., Patidar R., Rosenberg M., Ambrogio L., Auclair D., Wang J., Song Y.K. (2014). Comprehensive genomic analysis of rhabdomyosarcoma reveals a landscape of alterations affecting a common genetic axis in fusion-positive and fusion-negative tumors. Cancer Discov..

[16] Ognjanovic S., Linabery A.M., Charbonneau B., Ross J.A. (2009). Trends in childhood rhabdomyosarcoma incidence and survival in the United States, 1975–2005. Cancer.

[17] Williamson D., Missiaglia E., de Reynies A., Pierron G., Thuille B., Palenzuela G., Thway K., Orbach D., Laé M., Fréneaux P. (2010). Fusion Gene–Negative Alveolar Rhabdomyosarcoma Is Clinically and Molecularly Indistinguishable From Embryonal Rhabdomyosarcoma. J. Clin. Oncol..

[18] Davicioni E., Anderson M.J., Finckenstein F.G., Lynch J.C., Qualman S.J., Shimada H., Schofield D.E., Buckley J.D., Meyer W.H., Sorensen P.H. (2009). Molecular Classification of Rhabdomyosarcoma—Genotypic and Phenotypic Determinants of Diagnosis: A Report from the Children’s Oncology Group. Am. J. Pathol..

[19] Skapek S.X., Anderson J., Barr F.G., Bridge J.A., Gastier-Foster J.M., Parham D.M., Rudzinski E.R., Triche T., Hawkins D.S. (2013). *PAX-FOXO1* fusion status drives unfavorable outcome for children with rhabdomyosarcoma: A children’s oncology group report. Pediatr. Blood Cancer.

[20] Arnold M.A., Anderson J.R., Gastier-Foster J.M., Barr F.G., Skapek S.X., Hawkins D.S., Raney Jr R.B., Parham D.M., Teot L.A., Rudzinski E.R. (2016). Histology, Fusion Status, and Outcome in Alveolar Rhabdomyosarcoma With Low-Risk Clinical Features: A Report From the Children’s Oncology Group. Pediatr. Blood Cancer.

[21] Missiaglia E., Williamson D., Chisholm J., Wirapati P., Pierron G., Petel F., Concordet J.P., Thway K., Oberlin O., Pritchard-Jones K. (2012). *PAX3/FOXO1* Fusion Gene Status Is the Key Prognostic Molecular Marker in Rhabdomyosarcoma and Significantly Improves Current Risk Stratification. J. Clin. Oncol..

[22] Oberoi S., Crane J.N., Haduong J.H., Rudzinski E.R., Wolden S.L., Dasgupta R., Linardic C.M., Weiss A.R., Venkatramani R., Children’s Oncology Group Soft Tissue Sarcoma Committee (2023). Children’s Oncology Group’s 2023 blueprint for research: Soft tissue sarcomas. Pediatr. Blood Cancer.

[23] Hettmer S., Linardic C.M., Kelsey A., Rudzinski E.R., Vokuhl C., Selfe J., Ruhen O., Shern J.F., Khan J., Kovach A.R. (2022). Molecular testing of rhabdomyosarcoma in clinical trials to improve risk stratification and outcome: A consensus view from European paediatric Soft tissue sarcoma Study Group, Children’s Oncology Group and Cooperative Weichteilsarkom-Studiengruppe. Eur. J. Cancer.

[24] Gupta A.A., Xue W., Harrison D.J., Hawkins D.S., Dasgupta R., Wolden S., Shulkin B., Qumseya A., Routh J.C., MacDonald T. (2024). Addition of temsirolimus to chemotherapy in children, adolescents, and young adults with intermediate-risk rhabdomyosarcoma (ARST1431): A randomised, open-label, phase 3 trial from the Children’s Oncology Group. Lancet Oncol..

[25] Kubo T., Shimose S., Fujimori J., Furuta T., Ochi M. (2015). Prognostic value of PAX3/7-FOXO1 fusion status in alveolar rhabdomyosarcoma: Systematic review and meta-analysis. Crit. Rev. Oncol..

[26] Sorensen P.H., Lynch J.C., Qualman S.J., Tirabosco R., Lim J.F., Maurer H.M., Bridge J.A., Crist W.M., Triche T.J., Barr F.G. (2002). PAX3-FKHR and PAX7-FKHR gene fusions are prognostic indicators in alveolar rhabdomyosarcoma: A report from the Children’s Oncology Group. J. Clin. Oncol..

[27] Heske C.M., Chi Y.Y., Venkatramani R., Li M., Arnold M.A., Dasgupta R., Hiniker S.M., Hawkins D.S., Mascarenhas L. (2020). Survival Outcomes of Patients With Localized FOXO1 Fusion-Positive Rhabdomyosarcoma Treated on Recent Clinical Trials: A Report From the Soft Tissue Sarcoma Committee of the Children’s Oncology Group. Cancer March.

[28] Duan F., Smith L.M., Gustafson D.M., Zhang C., Dunlevy M.J., Gastier-Foster J.M., Barr F.G. (2012). Genomic and clinical analysis of fusion gene amplification in rhabdomyosarcoma: A report from the Children’s Oncology Group. Genes Chromosoes Cancer.

[29] Barr F.G., Duan F., Smith L.M., Gustafson D., Pitts M., Hammond S., Gastier-Foster J.M. (2009). Genomic and clinical analyses of 2p24 and 12q13-q14 amplification in alveolar rhabdomyosarcoma: A report from the children’s oncology group. Genes Chromosomes Cancer.

[30] Heinz A.T., Ciuffolotti M., Merks J.H., Schönstein A., Minard-Colin V., Fuchs J., Guillen G., Timmermann B., Vokuhl C., Koscielniak E. (2025). Treatment of Pediatric, Adolescent, and Young Adult Patients With Fusion-Positive Alveolar Rhabdomyosarcoma Infiltrating Regional Lymph Nodes in the European CWS-2002P and RMS 2005 Studies and the Soft Tissue Sarcoma Registry. Pediatr. Blood Cancer.

[31] Gallego S., Chi Y.Y., De Salvo G.L., Li M., Merks J.H., Rodeberg D.A., van Scheltinga S.T., Mascarenhas L., Orbach D., Jenney M. (2021). Alveolar rhabdomyosarcoma with regional nodal involvement: Results of a combined analysis from two cooperative groups. Pediatr. Blood Cancer.

[32] Huang S.C., Chen S., Kashikar S., Estilo C.L., Wolden S.L., Wexler L.H., Huryn J.M., Antonescu C.R. (2016). A clinicopathologic study of head and neck rhabdomyosarcomas showing FOXO1 fusion-positive alveolar and MYOD1-mutant sclerosing are associated with unfavorable outcome. Oral Oncol..

[33] Leiner J., Le Loarer F. (2020). The current landscape of rhabdomyosarcomas: An update. Virchows Arch..

[34] Shukla N., Ameur N., Yilmaz I., Nafa K., Lau C.Y., Marchetti A., Borsu L., Barr F.G., Ladanyi M. (2012). Oncogene mutation profiling of pediatric solid tumors reveals significant subsets of embryonal rhabdomyosarcoma and neuroblastoma with mutated genes in growth signaling pathways. Clin. Cancer Res..

[35] Chen X., Stewart E., Shelat A.A., Qu C., Bahrami A., Hatley M., Wu G., Bradley C., McEvoy J., Pappo A. (2013). Targeting Oxidative Stress in Embryonal Rhabdomyosarcoma. Cancer Cell.

[36] Seki M., Nishimura R., Yoshida K., Shimamura T., Shiraishi Y., Sato Y., Kato M., Chiba K., Tanaka H., Hoshino N. (2015). Integrated genetic and epigenetic analysis defines novel molecular subgroups in rhabdomyosarcoma. Nat. Commun..

[37] Song B., Yang P., Zhang S. (2024). Cell fate regulation governed by p53: Friends or reversible foes in cancer therapy. Cancer Commun..

[38] Guo Y., Wu H., Wiesmüller L., Chen M. (2024). Canonical and non-canonical functions of p53 isoforms: Potentiating the complexity of tumor development and therapy resistance. Cell Death Dis..

[39] Thoenen E., Curl A., Iwakuma T. (2019). TP53 in bone and soft tissue sarcomas. Pharmacol. Ther..

[40] Parrales A., Iwakuma T. (2015). Targeting oncogenic mutant p53 for cancer therapy. Front. Oncol..

[41] Lane D., Levine A. (2010). p53 Research: The past thirty years and the next thirty years. Cold Spring Harb. Perspect. Biol..

[42] Ranjan A., Iwakuma T. (2016). Non-canonical cell death induced by p53. Int. J. Mol. Sci..

[43] Adhikari A.S., Iwakuma T. (2009). Mutant p53 gain of oncogenic function: In vivo evidence, mechanism of action and its clinical implications. Fukuoka Igaku Zasshi Hukuoka Acta Med..

[44] Kim M.P., Lozano G. (2018). Mutant p53 partners in crime. Cell Death Differ..

[45] Mantovani F., Collavin L., Del Sal G. (2019). Mutant p53 as a guardian of the cancer cell. Cell Death Differ..

[46] Light N., Layeghifard M., Attery A., Subasri V., Zatzman M., Anderson N.D., Hatkar R., Blay S., Chen D., Novokmet A. (2023). Germline TP53 mutations undergo copy number gain years prior to tumor diagnosis. Nat. Commun..

[47] Haduong J.H., Heske C.M., Allen-Rhoades W., Xue W., Teot L.A., Rodeberg D.A., Donaldson S.S., Weiss A., Hawkins D.S., Venkatramani R. (2022). An update on rhabdomyosarcoma risk stratification and the rationale for current and future Children’s Oncology Group clinical trials. Pediatr. Blood Cancer.

[48] Shenoy A., Alvarez E., Chi Y.Y., Li M., Shern J.F., Khan J., Hiniker S.M., Granberg C.F., Hawkins D.S., Parham D.M. (2021). The prognostic significance of anaplasia in childhood rhabdomyosarcoma: A report from the Children’s Oncology Group. Eur. J. Cancer.

[49] Hettmer S., Archer N.M., Somers G.R., Novokmet A., Wagers A.J., Diller L., Rodriguez-Galindo C., Teot L.A., Malkin D. (2014). Anaplastic rhabdomyosarcoma in TP53 germline mutation carriers. Cancer.

[50] Villani A., Tabori U., Schiffman J., Shlien A., Beyene J., Druker H., Novokmet A., Finlay J., Malkin D. (2011). Biochemical and imaging surveillance in germline TP53 mutation carriers with Li-Fraumeni syndrome: A prospective observational study. Lancet Oncol..

[51] Goudie C., Coltin H., Witkowski L., Mourad S., Malkin D., Foulkes W.D. (2017). The McGill Interactive Pediatric OncoGenetic Guidelines: An approach to identifying pediatric oncology patients most likely to benefit from a genetic evaluation. Pediatr. Blood Cancer.

[52] Chisholm J.C., Selfe J.L., Alaggio R., Cheesman E., Zin A., Tombolan L., Parafioriti A., Milano G.M., Adams M., Popov S. (2025). Clinicopathological Analysis of a European Cohort of *MYOD1* Mutant Rhabdomyosarcomas in Children and Young Adults. Pediatr. Blood Cancer.

[53] Alaggio R., Zhang L., Sung Y.S., Huang S.C., Chen C.L., Bisogno G., Zin A., Agaram N.P., LaQuaglia M.P., Wexler L.H. (2016). A Molecular Study of Pediatric Spindle and Sclerosing Rhabdomyosarcoma: Identification of Novel and Recurrent VGLL2-related Fusions in Infantile Cases. Am. J. Surg. Pathol..

[54] Agaram N.P., Chen C.L., Zhang L., Laquaglia M.P., Wexler L., Antonescu C.R. (2014). Recurrent MYOD1 mutations in pediatric and adult sclerosing and spindle cell rhabdomyosarcomas: Evidence for a common pathogenesis. Genes Chromosomes Cancer.

[55] Kohsaka S., Shukla N., Ameur N., Ito T., Ng C.K., Wang L., Lim D., Marchetti A., Viale A., Pirun M. (2014). A recurrent neomorphic mutation in MYOD1 defines a clinically aggressive subset of embryonal rhabdomyosarcoma associated with PI3K-AKT pathway mutations. Nat. Genet..

[56] Agaram N.P., LaQuaglia M.P., Alaggio R., Zhang L., Fujisawa Y., Ladanyi M., Wexler L.H., Antonescu C.R. (2019). MYOD1-mutant spindle cell and sclerosing rhabdomyosarcoma: An aggressive subtype irrespective of age. A reappraisal for molecular classification and risk stratification. Mod. Pathol..

[57] Dehner C.A., Broski S.M., Meis J.M., Murugan P., Chrisinger J.S., Sosa C., Petersen M., Halling K.C., Gupta S., Folpe A.L. (2023). Fusion-driven Spindle Cell Rhabdomyosarcomas of Bone and Soft Tissue: A Clinicopathologic and Molecular Genetic Study of 25 Cases. Mod. Pathol..

[58] Schöpf J., Uhrig S., Heilig C.E., Lee K.S., Walther T., Carazzato A., Dobberkau A.M., Weichenhan D., Plass C., Hartmann M. (2024). Multi-omic and functional analysis for classification and treatment of sarcomas with FUS-TFCP2 or EWSR1-TFCP2 fusions. Nat. Commun..

[59] Fang Z., Duan C., Wang S., Fu L., Yang P., Yu T., Deel M.D., Lau L.M., Ma X., Ni X. (2024). Pediatric spindle cell/sclerosing rhabdomyosarcoma with FUS–TFCP2 fusion: A case report and literature review. Transl. Pediatr..

[60] Ginn M.P., Denu R.A., Ingram D.R., Wani K.M., Lazar A.J., Harrison D.J., Nakazawa M.S., Conley A.P., Patel S., Livingston J.A. (2025). TFCP2 Fusion-Positive Rhabdomyosarcomas: A Report of 10 Cases and a Review of the Literature. Cancers.

[61] Sivakumar N., Sharma P., Chandra S., Gupta S., Samadi F.M., Baghel S. (2023). Clinicopathological and Molecular Characteristics of Intraosseous Rhabdomyosarcoma Involving Head and Neck Region: A Systematic Review and Meta-Analysis. Pediatr. Dev. Pathol..

[62] Duan F.L., Yang H., Gong X., Zuo Z., Qin S., Ji J., Zhou C., Dai J., Guo P., Liu Y. (2023). Clinicopathological features of rhabdomyosarcoma with novel *FET::TFCP2* and *TIMP3::ALK* fusion: Report of two cases and literature review. Histopathology.

[63] Wong D.D., van Vliet C., Gaman A., Giardina T., Amanuel B. (2019). Rhabdomyosarcoma with FUS re-arrangement: Additional case in support of a novel subtype. Pathology.

[64] Csizmok V., Grisdale C.J., Williamson L.M., Lim H.J., Lee L., Renouf D.J., Jones S.J., Marra M.A., Laskin J., Smrke A. (2024). Diagnostic and Therapeutic Implications of a *FUS*::*TFCP2* Fusion and *ALK* Activation in a Metastatic Rhabdomyosarcoma. Genes Chromosomes Cancer.

[65] Schöpf J., Uhrig S., Heilig C.E., Lee K.S., Walther T., Carazzato A., Dobberkau A.M., Weichenhan D., Plass C., Hartmann M. (2023). Multidimensional Characterization of Soft-Tissue Sarcomas with FUS-TFCP2 or EWSR1-TFCP2 Fusions. bioRxiv.

[66] Han R., Dermawan J.K., Demicco E.G., Ferguson P.C., Griffin A.M., Swanson D., Antonescu C.R., Dickson B.C. (2022). ZFP64::NCOA3 gene fusion defines a novel subset of spindle cell rhabdomyosarcoma. Genes Chromosomes Cancer.

[67] Xu J., Wu R.C., O’Malley B.W. (2009). Normal and cancer-related functions of the p160 steroid receptor co-activator (SRC) family. Nat. Rev. Cancer.

[68] Gupta A., Hossain M.M., Miller N., Kerin M., Callagy G., Gupta S. (2016). NCOA3 coactivator is a transcriptional target of XBP1 and regulates PERK-eIF2α-ATF4 signalling in breast cancer. Oncogene.

[69] Li Y., Liang J., Dang H., Zhang R., Chen P., Shao Y. (2022). NCOA3 is a critical oncogene in thyroid cancer via the modulation of major signaling pathways. Endocrine.

[70] Sakamoto K., Tamamura Y., Katsube K.I., Yamaguchi A. (2008). Zfp64 participates in Notch signaling and regulates differentiation in mesenchymal cells. J. Cell Sci..

[71] Dermawan J.K., Malik F., Gross J.M., Baraban E., Pratilas C., Mneimneh W., Trucco M., Sun W., Barr F.G., Costa F.D.A. (2024). Novel PAX3::MAML3 Fusion Identified in Alveolar Rhabdomyosarcoma, Using DNA Methylation Profiling to Expand the Genetic Spectrum of “Fusion-Positive” Cases. Mod. Pathol..

[72] Joshi D., Anderson J.R., Paidas C., Breneman J., Parham D.M., Crist W. (2004). Age Is an Independent Prognostic Factor in Rhabdomyosarcoma: A Report from the Soft Tissue Sarcoma Committee of the Children’s Oncology Group. Pediatr. Blood Cancer.

[73] Bisogno G., Compostella A., Ferrari A., Pastore G., Cecchetto G., Garaventa A., Indolfi P., De Sio L., Carli M. (2012). Rhabdomyosarcoma in adolescents: A report from the AIEOP Soft Tissue Sarcoma Committee. Cancer.

[74] Ferrari A., Dileo P., Casanova M., Bertulli R., Meazza C., Gandola L., Navarria P., Collini P., Gronchi A., Olmi P. (2003). Rhabdomyosarcoma in adults: A retrospective analysis of 171 patients treated at a single institution. Cancer.

[75] Sultan I., Qaddoumi I., Yaser S., Rodriguez-Galindo C., Ferrari A. (2009). Comparing adult and pediatric rhabdomyosarcoma in the surveillance, epidemiology and end results program, 1973 to 2005: An analysis of 2600 patients. J. Clin. Oncol..

[76] Van Gaal J.C., Van der Graaf W.T., Rikhof B., Van Hoesel Q.G., Teerenstra S., Suurmeijer A.J., Flucke U.E., Loeffen J.L., Sleijfer S., De Bont E.S. (2012). The impact of age on outcome of embryonal and alveolar rhabdomyosarcoma patients. A multicenter study. Anticancer Res..

[77] Ferrari A., Gatz S.A., Minard-Colin V., Alaggio R., Hovsepyan S., Orbach D., Gasparini P., Defachelles A.S., Casanova M., Milano G.M. (2022). Shedding a Light on the Challenges of Adolescents and Young Adults with Rhabdomyosarcoma. Cancers.

[78] Ferrari A., Bernasconi A., Bergamaschi L., Botta L., Andreano A., Castaing M., Rugge M., Bisogno G., Falcini F., Sacerdote C. (2021). Impact of Rhabdomyosarcoma Treatment Modalities by Age in a Population-Based Setting. J. Adolesc. Young Adult Oncol..

[79] Bergamaschi L., Bertulli R., Casanova M., Provenzano S., Chiaravalli S., Gasparini P., Collini P., Sangalli C., Gandola L., Diletto B. (2019). Rhabdomyosarcoma in adults: Analysis of treatment modalities in a prospective single-center series. Med. Oncol..

[80] Ferrari A., Chisholm J.C., Jenney M., Minard-Colin V., Orbach D., Casanova M., Guillen G., Glosli H., van Rijn R.R., Schoot R.A. (2022). Adolescents and young adults with rhabdomyosarcoma treated in the European paediatric Soft tissue sarcoma Study Group (EpSSG) protocols: A cohort study. Lancet Child Adolesc. Health.

[81] Breneman J.C., Lyden E., Pappo A.S., Link M.P., Anderson J.R., Parham D.M., Qualman S.J., Wharam M.D., Donaldson S.S., Maurer H.M. (2003). Prognostic factors and clinical outcomes in children and adolescents with metastatic rhabdomyosarcoma-a report from the intergroup rhabdomyosarcoma study IV. J. Clin. Oncol..

[82] Oberlin O., Rey A., Lyden E., Bisogno G., Stevens M.C., Meyer W.H., Carli M., Anderson J.R. (2008). Prognostic Factors in Metastatic Rhabdomyosarcomas: Results of a Pooled Analysis From United States and European Cooperative Groups. J. Clin. Oncol..

[83] Chisholm J.C., Merks J.H., Casanova M., Bisogno G., Orbach D., Gentet J.C., Thomassin-Defachelles A.S., Chastagner P., Lowis S., Ronghe M. (2017). Open-label, multicentre, randomised, phase II study of the EpSSG and the ITCC evaluating the addition of bevacizumab to chemotherapy in childhood and adolescent patients with metastatic soft tissue sarcoma (the BERNIE study). Eur. J. Cancer.

[84] Hibbitts E., Chi Y.Y., Hawkins D.S., Barr F.G., Bradley J.A., Dasgupta R., Meyer W.H., Rodeberg D.A., Rudzinski E.R., Spunt S.L. (2019). Refinement of risk stratification for childhood rhabdomyosarcoma using FOXO1 fusion status in addition to established clinical outcome predictors: A report from the Children’s Oncology Group. Cancer Med..

[85] Mercolini F., Merks J.H., Minard-Colin V., Cameron A., van Scheltinga S.E.T., Sher O., Fichera G., Orbach D., Glosli H., Coppadoro B. (2023). Metastatic rhabdomyosarcoma with exclusive distant lymph node involvement: A European Pediatric Soft tissue sarcoma Study Group (EpSSG) report. Pediatr. Blood Cancer.

[86] Vasquez J.C., Luo L.Y., Hiniker S.M., Rhee D.S., Dasgupta R., Chen S., Weigel B.J., Xue W., Venkatramani R., Arndt C.A. (2023). Rhabdomyosarcoma with isolated lung metastases: A report from the Soft Tissue Sarcoma Committee of the Children’s Oncology Group. Pediatr. Blood Cancer.

[87] Chisholm J.C., Schoot R.A., Cameron A.L., Casanova M., Minard-Colin V., Coppadoro B., Garrido M., Rogers T., Orbach D., Glosli H. (2023). Outcomes in lung-only metastatic rhabdomyosarcoma: An analysis of data from the European paediatric Soft tissue sarcoma Study Group MTS 2008 study. EJC Paediatr. Oncol..

[88] Weigel B.J., Lyden E., Anderson J.R., Meyer W.H., Parham D.M., Rodeberg D.A., Michalski J.M., Hawkins D.S., Arndt C.A. (2016). Intensive multiagent therapy, including dose-compressed cycles of ifosfamide/etoposide and vincristine/doxorubicin/cyclophosphamide, irinotecan, and radiation, in patients with high-risk rhabdomyosarcoma: A report from the children’s oncology group. J. Clin. Oncol..

[89] La T.H., Wolden S.L., Rodeberg D.A., Hawkins D.S., Brown K.L., Anderson J.R., Donaldson S.S. (2011). Regional nodal involvement and patterns of spread along in-transit pathways in children with rhabdomyosarcoma of the extremity: A report from the children’s oncology group. Int. J. Radiat. Oncol. Biol. Phys..

[90] Terwisscha van Scheltinga S.E., Wijnen M.H., Martelli H., Rogers T., Mandeville H., Gaze M.N., McHugh K., Corradini N., Orbach D., Jenney M. (2020). Local staging and treatment in extremity rhabdomyosarcoma. A report from the EpSSG-RMS2005 study. Cancer Med..

[91] van Scheltinga C.T., Wijnen M.H.W.A., Martelli H., Guerin F., Rogers T., Craigie R.J., Burrieza G.G., Dall’Igna P., De Corti F., Smeulders N. (2022). In transit metastases in children, adolescents and young adults with localized rhabdomyosarcoma of the distal extremities: Analysis of the EpSSG RMS 2005 study. Eur. J. Surg. Oncol..

[92] Sparber-Sauer M., von Kalle T., Seitz G., Dantonello T., Scheer M., Münter M., Fuchs J., Ladenstein R., Bielack S.S., Klingebiel T. (2017). The prognostic value of early radiographic response in children and adolescents with embryonal rhabdomyosarcoma stage IV, metastases confined to the lungs: A report from the Cooperative Weichteilsarkom Studiengruppe (CWS). Pediatr. Blood Cancer.

[93] Wyatt K.D., Birz S., Hawkins D.S., Minard-Colin V., Rodeberg D.A., Sparber-Sauer M., Bisogno G., Koscielniak E., De Salvo G.L., Ebinger M. (2022). Creating a data commons: The INternational Soft Tissue SaRcoma ConsorTium (INSTRuCT). Pediatr. Blood Cancer.

[94] Di Carlo D., Affinita M.C., Poli E., Bisogno G. (2025). Systemic therapy in metastatic pediatric rhabdomyosarcoma: A history of challenges and the search for promising approaches. Expert Opin. Pharmacother..

[95] Maurer H.M., Crist W., Lawrence W., Ragab A.H., Raney R.B., Webber B., Wharam M., Vietti T.J., Beltangady M., Gehan E.A. (1988). The intergroup rhabdomyosarcoma study-I. A final report. Cancer.

[96] Maurer H.M., Gehan E.A., Beltangady M., Crist W., Dickman P.S., Donaldson S.S., Fryer C., Hammond D., Hays D.M., Herrmann J. (1993). The intergroup rhabdomyosarcoma study-II. Cancer.

[97] Crist W., Gehan E.A., Ragab A.H., Dickman P.S., Donaldson S.S., Fryer C., Hammond D., Hays D.M., Herrmann J., Heyn R. (1995). The Third Intergroup Rhabdomyosarcoma Study. J. Clin. Oncol..

[98] Pappo A.S., Lyden E., Breneman J., Wiener E., Teot L., Meza J., Crist W., Vietti T. (2001). Up-front window trial of topotecan in previously untreated children and adolescents with metastatic rhabdomyosarcoma: An intergroup rhabdomyosarcoma study. J. Clin. Oncol..

[99] Walterhouse D.O., Lyden E.R., Breitfeld P.P., Qualman S.J., Wharam M.D., Meyer W.H. (2004). Efficacy of topotecan and cyclophosphamide given in a phase II window trial in children with newly diagnosed metastatic rhabdomyosarcoma: A children’s oncology group study. J. Clin. Oncol..

[100] Pappo A.S., Lyden E., Breitfeld P., Donaldson S.S., Wiener E., Parham D., Crews K.R., Houghton P., Meyer W.H. (2007). Two consecutive phase II window trials of irinotecan alone or in combination with vincristine for the treatment of metastatic rhabdomyosarcoma: The children’s oncology group. J. Clin. Oncol..

[101] Arndt C.A., Stoner J.A., Hawkins D.S., Rodeberg D.A., Hayes-Jordan A.A., Paidas C.N., Parham D.M., Teot L.A., Wharam M.D., Breneman J.C. (2009). Vincristine, Actinomycin, and Cyclophosphamide Compared With Vincristine, Actinomycin, and Cyclophosphamide Alternating With Vincristine, Topotecan, and Cyclophosphamide for Intermediate-Risk Rhabdomyosarcoma: Children’s Oncology Group Study D9803. J. Clin. Oncol..

[102] Casey D.L., Chi Y.Y., Donaldson S.S., Hawkins D.S., Tian J., Arndt C.A., Rodeberg D.A., Routh J.C., Lautz T.B., Gupta A.A. (2019). Increased local failure for patients with intermediate-risk rhabdomyosarcoma on ARST0531: A report from the Children’s Oncology Group. Cancer.

[103] Malempati S., Weigel B.J., Chi Y.Y., Tian J., Anderson J.R., Parham D.M., Teot L.A., Rodeberg D.A., Yock T.I., Shulkin B.L. (2019). The addition of cixutumumab or temozolomide to intensive multiagent chemotherapy is feasible but does not improve outcome for patients with metastatic rhabdomyosarcoma: A report from the Children’s Oncology Group. Cancer.

[104] Carli M., Pastore G., Perilongo G., Grotto P., De Bernardi B., Ceci A., Di Tullio M., Madon E., Pianca C., Paolucci G. (1988). Tumor response and toxicity after single high-dose versus standard five-day divided-dose dactinomycin in childhood rhabdomyosarcoma. J. Clin. Oncol..

[105] Carli M., Colombatti R., Oberlin O., Bisogno G., Treuner J., Koscielniak E., Tridello G., Garaventa A., Pinkerton R., Stevens M. (2004). European intergroup studies (MMT4-89 and MMT4-91) on childhood metastatic rhabdomyosarcoma: Final results and analysis of prognostic factors. J. Clin. Oncol..

[106] Oberlin O., Rey A., Sanchez de Toledo J., Martelli H., Jenney M.E., Scopinaro M., Bergeron C., Merks J.H., Bouvet N., Ellershaw C. (2012). Randomized comparison of intensified six-drug versus standard three-drug chemotherapy for high-risk nonmetastatic rhabdomyosarcoma and other chemotherapy-sensitive childhood soft tissue sarcomas: Long-term results from the International Society of Pediatric Oncology MMT95 study. J. Clin. Oncol..

[107] McDowell H.P., Foot A.B.M., Ellershaw C., Machin D., Giraud C., Bergeron C. (2010). Outcomes in paediatric metastatic rhabdomyosarcoma: Results of The International Society of Paediatric Oncology (SIOP) study MMT-98. Eur. J. Cancer.

[108] Chisholm J.C., Merks J.H., Casanova M., Bisogno G., Orbach D., Gentet J.C., Thomassin Defachelles A.S., Chastagner P.B., Lowis S., Ronghe M. (2016). BERNIE: Open-label, randomized, phase II study of bevacizumab plus chemotherapy in pediatric metastatic rhabdomyosarcoma (RMS) and non-rhabdomyosarcoma soft tissue sarcoma (NRSTS). J. Clin. Oncol..

[109] Koscielniak E., Treuner J., Jürgens H., Winkler K., Bürger D., Herbst M., Ritter J., Niethammer D., Müller-Weihrich S., Bernhard G. (1991). Treatment of soft tissue sarcomas in children and adolescents. Results of the CWS-81 Multicenter Therapy Study. Klin. Padiatr..

[110] Koscielniak E., Harms D., Henze G., Jürgens H., Gadner H., Herbst M., Klingebiel T., Schmidt B.F., Morgan M., Knietig R. (1999). Results of treatment for soft tissue sarcoma in childhood and adolescence: A final report of the German cooperative soft tissue sarcoma study CWS-86. J. Clin. Oncol..

[111] Klingebiel T., Boos J., Beske F., Hallmen E., Int-Veen C., Dantonello T., Treuner J., Gadner H., Marky I., Kazanowska B. (2008). Treatment of children with metastatic soft tissue sarcoma with oral maintenance compared to high-dose chemotherapy: Report of the HD CWS-96 trial. Pediatr. Blood Cancer.

[112] Bisogno G., Pastore G., Perilongo G., Sotti G., Cecchetto G., Dallorso S., Carli M. (2012). Long-term results in childhood rhabdomyosarcoma: A report from the Italian Cooperative Study RMS 79. Pediatr. Blood Cancer.

[113] Raney R.B., Crist W.M., Maurer H.M., Foulkes M.A. (1983). Prognosis of children with soft tissue sarcoma who relapse after achieving a complete response. A report from the Intergroup Rhabdomyosarcoma Study I. Cancer.

[114] Raney R.B., Maurer H.M., Anderson J.R., Andrassy R.J., Donaldson S.S., Qualman S.J., Wharam M.D., Wiener E.S., Crist W.M. (2001). The Intergroup Rhabdomyosarcoma Study Group (IRSG): Major lessons from the IRS-I through IRS-IV studies as background for the current IRS-V treatment protocols. Sarcoma.

[115] Breitfeld P.P., Lyden E., Raney R.B., Teot L.A., Wharam M., Lobe T., Crist W.M., Maurer H.M., Donaldson S.S., Ruymann F.B. (2001). Ifosfamide and Etoposide Are Superior to Vincristine and Melphalan for Pediatric Metastatic Rhabdomyosarcoma When Administered With Irradiation and Combination Chemotherapy: A Report From the Intergroup Rhabdomyosarcoma Study Group. J. Pediatr. Hematol..

[116] Admiraal R., van der Paardt M., Kobes J., Kremer L.C., Bisogno G., Merks J.H. (2010). High-dose chemotherapy for children and young adults with stage IV rhabdomyosarcoma. Cochrane Database Syst. Rev..

[117] Bisogno G., Ferrari A., Prete A., Messina C., Basso E., Cecchetto G., Indolfi P., Scarzello G., D’Angelo P., De Sio L. (2009). Sequential high-dose chemotherapy for children with metastatic rhabdomyosarcoma. Eur. J. Cancer.

[118] Bisogno G., Hawkins D.S. (2020). An unresolved issue in rhabdomyosarcoma treatment: The duration of chemotherapy. Pediatr. Blood Cancer.

[119] Tramsen L., Bochennek K., Sparber-Sauer M., Salzmann-Manrique E., Scheer M., Dantonello T., Borkhardt A., Dirksen U., Thorwarth A., Greiner J. (2023). Pediatric Patients with Stage IV Rhabdomyosarcoma Significantly Benefit from Long-Term Maintenance Therapy: Results of the CWS-IV 2002 and the CWS DOK IV 2004-Trials. Cancers.

[120] Bisogno G., Minard-Colin V., Jenney M., Ferrari A., Chisholm J., Di Carlo D., Hjalgrim L.L., Orbach D., Merks J.H.M., Casanova M. (2023). Maintenance Chemotherapy for Patients with Rhabdomyosarcoma. Cancers.

[121] Bisogno G., De Salvo G.L., Bergeron C., Melcón S.G., Merks J.H., Kelsey A., Martelli H., Minard-Colin V., Orbach D., Glosli H. (2019). Vinorelbine and continuous low-dose cyclophosphamide as maintenance chemotherapy in patients with high-risk rhabdomyosarcoma (RMS 2005): A multicentre, open-label, randomised, phase 3 trial. Lancet Oncol..

[122] Dávila Fajardo R., Magelssen H., Cameron A.L., Boterberg T., Mandeville H.C. (2025). Current and Future Developments in Radiation Oncology Approach for Rhabdomyosarcoma. Cancers.

[123] Cameron A.L., Elze M.C., Casanova M., Geoerger B., Gaze M.N., Minard-Colin V., McHugh K., van Rijn R.R., Kelsey A., Martelli H. (2021). The Impact of Radiation Therapy in Children and Adolescents With Metastatic Rhabdomyosarcoma. Int. J. Radiat. Oncol. Biol. Phys..

[124] Cameron A.L., Mandeville H., Coppadoro B., Periasamy M., Fajardo R.D., Ferrari A., Gaze M.N., Helfre S., Magelssen H., Minard-Colin V. (2025). The Impact of Radiation Therapy on Metastatic Rhabdomyosarcoma: Results From the EpSSG MTS 2008 Study. Int. J. Radiat. Oncol. Biol. Phys..

[125] Ferrari A., Bergamaschi L., Chiaravalli S., Livellara V., Sironi G., Nigro O., Puma N., Gattuso G., Morosi C., Gasparini P. (2022). Metastatic rhabdomyosarcoma: Evidence of the impact of radiotherapy on survival. A retrospective single-center experience. Pediatr. Blood Cancer.

[126] Roy Moulik N., Vaidya S., Mandeville H., Chisholm J.C. (2019). Managing peritoneal involvement in children and young people with rhabdomyosarcoma: A single-center experience from the United Kingdom. Pediatr. Blood Cancer.

[127] Gesche J., Beckert S., Neunhoeffer F., Kachanov D., Königsrainer A., Seitz G., Fuchs J. (2019). Cytoreductive surgery and hyperthermic intraperitoneal chemotherapy: A safe treatment option for intraperitoneal rhabdomyosarcoma in children below 5 years of age. Pediatr. Blood Cancer.

[128] Hayes-Jordan A., Green H., Lin H., Owusu-Agyemang P., Mejia R., Okhuysen-Cawley R., Cortes J., Fitzgerald N.E., McAleer M.F., Herzog C. (2015). Cytoreductive Surgery and Hyperthermic Intraperitoneal Chemotherapy (HIPEC) for Children, Adolescents, and Young Adults: The First 50 Cases. Ann. Surg. Oncol..

[129] Byrwa D.J., Twist C.J., Skitzki J., Repasky E., Ham P.B.e.n., Gupta A. (2023). A Review of the Use of Hyperthermic Intraperitoneal Chemotherapy for Peritoneal Malignancy in Pediatric Patients. Cancers.

[130] Searcy M.B., Larsen R.K., Stevens B.T., Zhang Y., Jin H., Drummond C.J., Langdon C.G., Gadek K.E., Vuong K., Reed K.B. (2023). PAX3-FOXO1 dictates myogenic reprogramming and rhabdomyosarcoma identity in endothelial progenitors. Nat. Commun..

[131] Hoshino A., Costa-Silva B., Shen T.L., Rodrigues G., Hashimoto A., Tesic Mark M., Molina H., Kohsaka S., Di Giannatale A., Ceder S. (2015). Tumour exosome integrins determine organotropic metastasis. Nature.

[132] Kohama I., Kosaka N., Chikuda H., Ochiya T. (2019). An insight into the roles of microRNAs and exosomes in sarcoma. Cancers.

[133] Junttila M.R., De Sauvage F.J. (2013). Influence of tumour micro-environment heterogeneity on therapeutic response. Nature.

[134] Majmundar A.J., Wong W.J., Simon M.C. (2010). Hypoxia-Inducible Factors and the Response to Hypoxic Stress. Mol. Cell.

[135] Bernauer C., Man Y.K.S., Chisholm J.C., Lepicard E.Y., Robinson S.P., Shipley J.M. (2021). Hypoxia and its therapeutic possibilities in paediatric cancers. Br. J. Cancer.

[136] Petragnano F., Pietrantoni I., Camero S., Codenotti S., Milazzo L., Vulcano F., Macioce G., Giordani I., Tini P., Cheleschi S. (2020). Clinically relevant radioresistant rhabdomyosarcoma cell lines: Functional, molecular and immune-related characterization. J. Biomed. Sci..

[137] Klega K., Imamovic-Tuco A., Ha G., Clapp A.N., Meyer S., Ward A., Clinton C., Nag A., Van Allen E., Mullen E. (2018). Detection of Somatic Structural Variants Enables Quantification and Characterization of Circulating Tumor DNA in Children With Solid Tumors. JCO Precis. Oncol..

[138] Abbou S., Klega K., Tsuji J., Tanhaemami M., Hall D., Barkauskas D.A., Krailo M.D., Cibulskis C., Nag A., Thorner A.R. (2023). Circulating Tumor DNA Is Prognostic in Intermediate-Risk Rhabdomyosarcoma: A Report from the Children’s Oncology Group. J. Clin. Oncol..

[139] Heske C.M., Mascarenhas L. (2021). Relapsed rhabdomyosarcoma. J. Clin. Med..

[140] Defachelles A.S., Bogart E., Casanova M., Merks H., Bisogno G., Calareso G., Gallego Melcon S., Gatz S., Le Deley M.C., McHugh K. (2019). Randomized phase 2 trial of the combination of vincristine and irinotecan with or without temozolomide, in children and adults with refractory or relapsed rhabdomyosarcoma (RMS). J. Clin. Oncol..

[141] Mascarenhas L., Lyden E.R., Breitfeld P.P., Walterhouse D.O., Donaldson S.S., Rodeberg D.A., Parham D.M., Anderson J.R., Meyer W.H., Hawkins D.S. (2019). Risk-based treatment for patients with first relapse or progression of rhabdomyosarcoma: A report from the Children’s Oncology Group. Cancer.

[142] Chisholm J.C., Marandet J., Rey A., Scopinaro M., de Toledo J.S., Merks J.H., O‘Meara A., Stevens M.C., Oberlin O. (2011). Prognostic factors after relapse in nonmetastatic rhabdomyosarcoma: A nomogram to better define patients who can be salvaged with further therapy. J. Clin. Oncol..

[143] Smith L.M., Anderson J.R., Qualman S.J., Crist W.M., Paidas C.N., Teot L.A., Pappo A.S., Link M.P., Grier H.E., Wiener E.S. (2001). Which patients with microscopic disease and rhabdomyosarcoma experience relapse after therapy? A report from the Soft Tissue Sarcoma Committee of the Children’s Oncology Group. J. Clin. Oncol..

[144] Mazzoleni S., Bisogno G., Garaventa A., Cecchetto G., Ferrari A., Sotti G., Donfrancesco A., Madon E., Casula L., Carli M. (2005). Outcomes and prognostic factors after recurrence in children and adolescents with nonmetastatic rhabdomyosarcoma. Cancer.

[145] Dantonello T.M., Int-Veen C., Winkler P., Leuschner I., Schuck A., Schmidt B.F., Lochbuehler H., Kirsch S., Hallmen E., Veit-Friedrich I. (2008). Initial patient characteristics can predict pattern and risk of relapse in localized rhabdomyosarcoma. J. Clin. Oncol..

[146] Affinita M.C., Ferrari A., Chiaravalli S., Melchionda F., Quaglietta L., Casanova M., Zanetti I., Scarzello G., Di Pasquale L., Di Cataldo A. (2020). Defining the impact of prognostic factors at the time of relapse for nonmetastatic rhabdomyosarcoma. Pediatr. Blood Cancer.

[147] Vaarwerk B., Mallebranche C., Affinita M.C., van der Lee J.H., Ferrari A., Chisholm J.C., Defachelles A.S., De Salvo G.L., Corradini N., Minard-Colin V. (2020). Is surveillance imaging in pediatric patients treated for localized rhabdomyosarcoma useful? The European experience. Cancer.

[148] Rodeberg D.A., Garcia-Henriquez N., Lyden E.R., Davicioni E., Parham D.M., Skapek S.X., Hayes-Jordan A.A., Donaldson S.S., Brown K.L., Triche T.J. (2011). Prognostic significance and tumor biology of regional lymph node disease in patients with rhabdomyosarcoma: A report from the Children’s Oncology Group. J. Clin. Oncol..

[149] Ferrari A., Casanova M., Bisogno G., Zanetti I., Cecchetto G., Bernardi B.D., Riccardi R., Tamaro P., Meazza C., Alaggio R. (2003). Rhabdomyosarcoma in infants younger than one year old: A report from the Italian Cooperative Group. Cancer.

[150] Sparber-Sauer M., Stegmaier S., Vokuhl C., Seitz G., von Kalle T., Scheer M., Münter M., Bielack S.S., Weclawek-Tompol J., Ladenstein R. (2019). Rhabdomyosarcoma diagnosed in the first year of life: Localized, metastatic, and relapsed disease. Outcome data from five trials and one registry of the Cooperative Weichteilsarkom Studiengruppe (CWS). Pediatr. Blood Cancer.

[151] Malempati S., Rodeberg D.A., Donaldson S.S., Lyden E.R., Anderson J.R., Hawkins D.S., Arndt C.A.S. (2011). Rhabdomyosarcoma in infants younger than 1 year: A report from the children’s oncology group. Cancer.

[152] Bradley J.A., Kayton M.L., Chi Y.Y., Hawkins D.S., Tian J., Breneman J., Wolden S.L., Walterhouse D., Rodeberg D.A., Donaldson S.S. (2019). Treatment Approach and Outcomes in Infants With Localized Rhabdomyosarcoma: A Report From the Soft Tissue Sarcoma Committee of the Children’s Oncology Group. Int. J. Radiat. Oncol. Biol. Phys..

[153] Ragab A.H., Heyn R., Tefft M., Hays D.N., Newton W.A., Beltangady M. (1986). Infants younger than 1 year of age with rhabdomyosarcoma. Cancer.

[154] Mascarenhas L., Lyden E.R., Breitfeld P.P., Walterhouse D.O., Donaldson S.S., Paidas C.N., Parham D.M., Anderson J.R., Meyer W.H., Hawkins D.S. (2010). Randomized phase II window trial of two schedules of irinotecan with vincristine in patients with first relapse or progression of rhabdomyosarcoma: A report from the Children’s Oncology Group. J. Clin. Oncol..

[155] Mascarenhas L., Chi Y.Y., Hingorani P., Anderson J.R., Lyden E.R., Rodeberg D.A., Indelicato D.J., Kao S.C., Dasgupta R., Spunt S.L. (2019). Randomized phase II trial of bevacizumab or temsirolimus in combination with chemotherapy for first relapse rhabdomyosarcoma: A report from the Children’s Oncology Group. J. Clin. Oncol..

[156] Casanova M., Bautista F., Campbell-Hewson Q., Makin G., Marshall L.V., Verschuur A.C., Cañete Nieto A., Corradini N., Ploeger B.A., Brennan B.J. (2023). Regorafenib plus Vincristine and Irinotecan in Pediatric Patients with Recurrent/Refractory Solid Tumors: An Innovative Therapy for Children with Cancer Study. Clin. Cancer Res..

[157] Metts J., Xue W., Gao Z., Oberoi S., Weiss A.R., Venkatramani R., Harrison D.J. (2024). Event-free survival in relapsed and refractory rhabdomyosarcoma treated on cooperative group phase II trials: A report from the Children’s Oncology Group. Pediatr. Blood Cancer.

[158] Parsons D.W., Janeway K.A., Patton D.R., Winter C.L., Coffey B., Williams P.M., Roy-Chowdhuri S., Tsongalis G.J., Routbort M., Ramirez N.C. (2022). Actionable Tumor Alterations and Treatment Protocol Enrollment of Pediatric and Young Adult Patients with Refractory Cancers in the National Cancer Institute-Children’s Oncology Group Pediatric MATCH Trial. J. Clin. Oncol..

[159] Evans C., Shepherd L., Bryan G., Fulbright H., Crowther S., Wakeling S., Stewart A., Stewart C., Chisholm J., Gibson F. (2024). A systematic review of early phase studies for children and young people with relapsed and refractory rhabdomyosarcoma: The REFoRMS-SR project. Int. J. Cancer.

[160] Defachelles A.S., Breunis W.B., Casanova M., Minard-Collin V., Hjadun R., Wasti A., Fajardo R.D., van Scheltinga S.T., Heenen D., Heinz A.T. (2025). Relapsed rhabdomyosarcoma: Treatment recommendations from the European pediatric soft tissue sarcoma study group (EpSSG). Br. J. Cancer.

[161] Heinz A.T., Ebinger M., Schönstein A., Fuchs J., Timmermann B., Seitz G., Vokuhl C., Münter M.W., Pajtler K.W., Stegmaier S. (2023). Second-line treatment of pediatric patients with relapsed rhabdomyosarcoma adapted to initial risk stratification: Data of the European Soft Tissue Sarcoma Registry (SoTiSaR). Pediatr. Blood Cancer.

[162] Mattke A.C., Bailey E.J., Schuck A., Dantonello T., Leuschner I., Klingebiel T., Treuner J., Koscielniak E. (2009). Does the time-point of relapse influence outcome in pediatric rhabdomyosarcomas?. Pediatr. Blood Cancer.

[163] McCollough C.H., Rajendran K., Leng S., Yu L., Fletcher J.G., Stierstorfer K., Flohr T.G. (2023). The technical development of photon-counting detector CT. Eur. Radiol..

[164] Sawall S., Amato C., Klein L., Wehrse E., Maier J., Kachelrieß M. (2021). Toward molecular imaging using spectral photon-counting computed tomography?. Curr. Opin. Chem. Biol..

[165] Alberts I., Sari H., Mingels C., Afshar-Oromieh A., Pyka T., Shi K., Rominger A. (2023). Long-axial field-of-view PET/CT: Perspectives and review of a revolutionary development in nuclear medicine based on clinical experience in over 7000 patients. Cancer Imaging.

[166] Dimitrakopoulou-Strauss A., Pan L., Sachpekidis C. (2023). Long axial field of view (LAFOV) PET-CT: Implementation in static and dynamic oncological studies. Eur. J. Nucl. Med. Mol. Imaging.

[167] Borgwardt L., Brok J., Andersen K.F., Madsen J., Gillings N., Fosbøl M.Ø., Denholt C.L., Petersen I.N., Sørensen L.S., Enevoldsen L.H. (2024). Performing [^18^F]MFBG Long–Axial-Field-of-View PET/CT Without Sedation or General Anesthesia for Imaging of Children with Neuroblastoma. J. Nucl. Med..

[168] Crane J.N., Graham D.S., Mona C.E., Nelson S.D., Samiei A., Dawson D.W., Dry S.M., Masri M.G., Crompton J.G., Benz M.R. (2023). Fibroblast Activation Protein Expression in Sarcomas. Sarcoma.

[169] Giammarile F., Knoll P., Paez D., Estrada Lobato E., Calapaquí Terán A.K., Delgado Bolton R.C. (2024). Fibroblast Activation Protein Inhibitor (FAPI) PET Imaging in Sarcomas: A New Frontier in Nuclear Medicine. Semin. Nucl. Med..

[170] Gu B., Liu X., Wang S., Xu X., Liu X., Hu S., Yan W., Luo Z., Song S. (2022). Head-to-head evaluation of [18F]FDG and [68 Ga]Ga-DOTA-FAPI-04 PET/CT in recurrent soft tissue sarcoma. Eur. J. Nucl. Med. Mol. Imaging.

[171] van Ewijk R., Vaarwerk B., Breunis W.B., Schoot R.A., Ter Horst S.A., van Rijn R.R., van der Lee J.H., Merks J.H. (2021). The value of early tumor size response to chemotherapy in pediatric rhabdomyosarcoma. Cancers.

[172] Vaarwerk B., van der Lee J.H., Breunis W.B., Orbach D., Chisholm J.C., Cozic N., Jenney M., van Rijn R.R., McHugh K., Gallego S. (2018). Prognostic relevance of early radiologic response to induction chemotherapy in pediatric rhabdomyosarcoma: A report from the International Society of Pediatric Oncology Malignant Mesenchymal Tumor 95 study. Cancer.

[173] Hawkins C.M., Gill A.E. (2023). Introduction to interventional radiology’s role in palliative care for children with cancer: A COG Diagnostic Imaging Committee/SPR Oncology Committee White Paper. Pediatr. Blood Cancer.

[174] Tomasian A., Filippiadis D.K., Tutton S., Deschamps F., Cazzato R.L., Prologo J.D., Kelekis A., Levy J., Gangi A., Garnon J. (2022). Comprehensive Palliative Musculoskeletal Interventional Radiology Care for Patients with Cancer. Radiographics.

[175] Yun J.H., Fang A., Khorshidi F., Habibollahi P., Kutsenko O., Etezadi V., Hunt S., Nezami N. (2023). New Developments in Image-Guided Percutaneous Irreversible Electroporation of Solid Tumors. Curr. Oncol. Rep..

[176] Kim H.S., Chapiro J., Geschwind J.F.H. (2016). From the Guest Editor: Interventional Oncology: The Fourth Pillar of Oncology. Cancer J..

[177] Patel P.A., Muñoz F.G. (2024). Interventional oncology in children: Where are we now?. J. Med. Imaging Radiat. Oncol..

[178] Gómez F.M., Aguado A., Barnacle A.M., Runge J.H., Temple M. (2024). Opportunities for interventional radiology in paediatric oncology. EJC Paediatr. Oncol..

[179] Roebuck D.J. (2010). Paediatric interventional oncology. Cancer Imaging.

[180] van Ewijk R., Schoot R.A., Sparber-Sauer M., Ter Horst S.A., Jehanno N., Borgwardt L., de Keizer B., Merks J.H., de Luca A., McHugh K. (2021). European guideline for imaging in paediatric and adolescent rhabdomyosarcoma—Joint statement by the European Paediatric Soft Tissue Sarcoma Study Group, the Cooperative Weichteilsarkom Studiengruppe and the Oncology Task Force of the European Society of Paediatric Radiology. Pediatr. Radiol..

[181] Lak N.S., van Zogchel L.M., Zappeij-Kannegieter L., Javadi A., Van Paemel R., Vandeputte C., De Preter K., De Wilde B., Chicard M., Iddir Y. (2023). Cell-Free DNA as a Diagnostic and Prognostic Biomarker in Pediatric Rhabdomyosarcoma. JCO Precis. Oncol..

[182] Ruhen O., Lak N.S., Stutterheim J., Danielli S.G., Chicard M., Iddir Y., Saint-Charles A., Di Paolo V., Tombolan L., Gatz S.A. (2022). Molecular Characterization of Circulating Tumor DNA in Pediatric Rhabdomyosarcoma: A Feasibility Study. JCO Precis. Oncol..

[183] Bettegowda C., Sausen M., Leary R.J., Kinde I., Wang Y., Agrawal N., Bartlett B.R., Wang H., Luber B., Alani R.M. (2014). Detection of Circulating Tumor DNA in Early-and Late-Stage Human Malignancies. Sci. Transl. Med..

[184] Siravegna G., Mussolin B., Venesio T., Marsoni S., Seoane J., Dive C., Papadopoulos N., Kopetz S., Corcoran R.B., Siu L.L. (2019). How liquid biopsies can change clinical practice in oncology. Ann. Oncol..

[185] Chicard M., Colmet-Daage L., Clement N., Danzon A., Bohec M., Bernard V., Baulande S., Bellini A., Deveau P., Pierron G. (2018). Whole-exome sequencing of cell-free DNA reveals temporo-spatial heterogeneity and identifies treatment-resistant clones in neuroblastoma. Clin. Cancer Res..

[186] Lak N.S., Voormanns T.L., Zappeij-Kannegieter L., van Zogchel L.M., Fiocco M., van Noesel M.M., Merks J.H., van der Schoot C.E., Tytgat G.A., Stutterheim J. (2021). Improving Risk Stratification for Pediatric Patients with Rhabdomyosarcoma by Molecular Detection of Disseminated Disease. Clin. Cancer Res..

[187] Yohe M.E., Heske C.M., Stewart E., Adamson P.C., Ahmed N., Antonescu C.R., Chen E., Collins N., Ehrlich A., Galindo R.L. (2019). Insights into pediatric rhabdomyosarcoma research: Challenges and goals. Pediatr. Blood Cancer.

[188] Van Erp A.E.M., Versleijen-Jonkers Y.M.H., Van Der Graaf W.T.A., Fleuren E.D.G. (2018). Targeted therapy-based combination treatment in rhabdomyosarcoma. Mol. Cancer Ther..

[189] Fleuren E.D.G., Terry R.L., Meyran D., Omer N., Trapani J.A., Haber M., Neeson P.J., Ekert P.G. (2021). Enhancing the Potential of Immunotherapy in Paediatric Sarcomas: Breaking the Immunosuppressive Barrier with Receptor Tyrosine Kinase Inhibitors. Biomedicines.

[190] Majzner R.G., Rietberg S.P., Labanieh L., Sotillo E., Weber E.W., Lynn R.C., Theruvath J.L., Yuan C.M., Xu P., Nguyen S.M. (2018). Low CD19 Antigen Density Diminishes Efficacy of CD19 CAR T Cells and Can be Overcome By Rational Redesign of CAR Signaling Domains. Blood.

[191] Anderson J. (2017). Unleashing the immune response against childhood solid cancers. Pediatr. Blood Cancer.

[192] Dean A.Q., Luo S., Twomey J.D., Zhang B. (2021). Targeting cancer with antibody-drug conjugates: Promises and challenges. MAbs.

[193] Olsen H.E., Lynn G.M., Valdes P.A., Cerecedo Lopez C.D., Ishizuka A.S., Arnaout O., Bi W.L., Peruzzi P.P., Chiocca E.A., Friedman G.K. (2021). Therapeutic cancer vaccines for pediatric malignancies: Advances, challenges, and emerging technologies. Neuro-Oncol. Adv..

[194] Chen C., Dorado Garcia H., Scheer M., Henssen A.G. (2019). Current and Future Treatment Strategies for Rhabdomyosarcoma. Front. Oncol..

